# Service process factors affecting patients’ and clinicians’ experiences on rapid teleconsultation implementation in out-patient neurology services during COVID-19 pandemic: a scoping review

**DOI:** 10.1186/s12913-022-07908-4

**Published:** 2022-04-22

**Authors:** Guangxia Meng, Carrie McAiney, Christopher M. Perlman, Ian McKillop, Therese Tisseverasinghe, Helen H. Chen

**Affiliations:** 1 School of Public Health Sciences, University of Waterloo, 200 University Ave W, Waterloo, ON N2L 3G1 Canada; 2 Seneca Libraries, Seneca College, 13990 Dufferin St, King City, ON L7B 1B3 Canada

**Keywords:** Neurology, Teleconsultation, COVID 19, Outpatient, Service quality, Patient experience, Clinician perception

## Abstract

**Background:**

The adoption of teleconsultation for outpatient neurology services was limited until the onset of the COVID-19 pandemic which forced many outpatient neurology services to rapidly switch to virtual models. However, it remains unclear how this change has impacted patients’ and clinicians’ perceptions of service quality. The purpose of this scoping review is to identify process factors that influence patients’ and clinicians’ experiences of outpatient teleconsultation services during COVID-19.

**Methods:**

Arksey and O’Malley scoping review framework was used to search PubMed, Scopus, CINAHL, and PsycInfo for original peer-reviewed research studies that examined the experiences of synchronous teleconsultation between a clinician and patient in a home-setting since the World Health Organization announced the COVID-19 global pandemic. The service quality model SERVQUAL was used to conduct a deductive thematic analysis to identify the key factors that impacted the patients’ and clinicians’ perception of teleconsultation services.

**Results:**

A total of nineteen studies published between January 1, 2020, and April 17, 2021, were identified. The most common service process factors affecting the patients’ and clinicians’ experiences of teleconsultation were technical issues, addressing logistical needs, communication, ability to perform clinical activities, appropriate triage, and administrative support.

**Conclusions:**

Our findings identified six key service process factors affecting the patients’ and clinicians’ teleconsultation experiences in outpatient neurology services. The need for improvement of triage process and standardizing administrative virtual care pathway are identified as important steps to improve patients and clinicians’ teleconsultation experiences compared to pre-COVID era. More research is needed to assess outpatient neurology teleconsultation service quality from patients’ and clinicians’ perspectives.

**Supplementary Information:**

The online version contains supplementary material available at 10.1186/s12913-022-07908-4.

## Background

With approximately 3.6 million Canadians currently suffering from neurological conditions access to neurology-specific care has become an urgent need within the healthcare system [1]. Currently, the use of telemedicine in neurology is still in its infancy [2]. Before COVID-19, the most notable application of teleconsultation in neurology was telestroke, which provides acute stroke management care in underserved communities [3, 4]. However, the utilization of teleconsultation in other areas of neurology is not as clear [5]. Research in synchronous telemedicine for outpatient services is imitated to studies mainly in solving access issues, follow-up patients or patients with a confirmed diagnosis, or in a satellite clinic setting [6–11]. A Canadian pilot project initiated by Kingston Health Science Center (KHSC) stroke prevention clinic in August 2018 evaluated teleconsultation with stroke patients in a home setting. However, the virtual visits were exclusively for follow-up clinical activities such as reviewing investigations, symptoms management, and medication titrations [1]. Additionally, two separate clinical trials were published much earlier, examining the safety and feasibility of teleconsultation in new but non-urgent neurological patients [12, 13]. However, in both studies, the patients were consulted from a satellite clinic with a healthcare professional as the telepresenter to facilitate examination and demonstrate findings [12, 13]. The significant barriers to a large-scale adaptation of teleconsultation in outpatient neurology might be due to a lack of evidence for its efficacy and understanding of the proper place of teleconsultation in traditional practice [2]. There is no publication on teleconsultation regarding new patient referrals from a home setting prior to the COVID outbreak.

Traditional face-to-face consultation is a cornerstone of neurology practice. Thus, in-person visits during the COVID-19 pandemic have been deemed both unwise and unsafe [14]. The rapid altered outpatient service delivery included deferred elective visits, modified face-to-face consultations, and increased use of teleconsultation since the COVID-19 pandemic [15]. For example, the outpatient neurology consultations for multiple sclerosis (MS) and neuromyelitis optica spectrum disorders (NMOSD) decreased by approximately 50% during COVID-19 in Argentina, Chile, Colombia, and Brazil [16]. COVID-19 pandemic has catalyzed telemedicine in outpatient neurology specialties, as evidenced by how quickly many neurology clinics implemented some forms of teleconsultation [17]. For instance, since the onset of the pandemic, only 8% of Norway’s hospital-based neurologists maintained regular in-person visits in their outpatient clinics, while 87% of their colleagues shifted to telemedicine [18]. Similarly, an outpatient neurology clinic in a large academic medical center in the United States converted more than 90% of its in-person visits to telemedicine since the start of the COVID-19 outbreak [19]. A global survey involving 40 countries on telemedicine utilization for movement disorders between March and April 2020 indicated a global increase in telemedicine usage among all surveyed countries, even those with little or no prior use [20]. For instance, only 19.4% of neurologists in Latin American countries had experience using telemedicine before COVID-19, whereas, between March and July 2020, 79.8% were using this technology [16]. The rapid expansion of teleconsultation in outpatient neurology services during the COVID-19 pandemic occurred within a few weeks or even days, and has since permeated into various sub-specialties in neurology, offering patients access to care virtually from their homes, or from anywhere with an internet connection using their mobile devices.

The rapid expansion of teleconsultation in outpatient neurology care occurring without clear scientific evidence to guide this change could result in diminished service quality. Healthcare service quality is complex due to its intangible, heterogeneous, and subjective characteristics in some aspects [21]. With the current shift towards “person-centeredness” healthcare, this review will identify the service quality process factors from clinicians’ and patients’ experiences [22]. By focusing on clinicians’ and patients’ experiences in teleconsultation, we align with the quadruple aim framework, which specifies the following four principles: enhancing patient experiences, improving population health, reducing cost, and improving the work-life of health care providers [23]. Our review focuses on assessing the teleconsultation’s service process factors that affect clinicians’ and patients’ experiences during the rapid change of service delivery model in outpatient neurology during COVID-19. Process factors in service quality are all the acts of caregiving, such as diagnosis, treatment, and patient interactions [24], which are also relevant factors in assessing teleconsultation services. The most widely used process-orientated approach is the service quality (SERVQUAL) model [25]. The SERVQUAL model includes five dimensions: tangibles (the appearance of physical facilities, equipment, and personnel), reliability (the ability to perform the promised service dependably and accurately), responsiveness (the willingness to help customers and provide prompt service), assurance (the knowledge and courtesy of employees and their ability to inspire trust and confidence), and empathy (the provision of individual care and attention to customers) [26, 27]. There are altogether 22 service attributes listed within the five service dimensions in the SERVQUAL model, which can be adapted to fit the characteristics of a particular service [28]. An additional table shows this in more detail (see Additional file 1). We consider attributes as the process factors in our review. There are many instances of varying uses of the SERVQUAL model to assess service quality in telemedicine. For example, the SERVQUAL questionnaire was used to assess the service quality of a telehealth program in a hospital setting [29]. The theory-driven analysis allows the researcher to identify the service process factors, reveal existing predispositions about study results, and assist in data coding and interpretation [30].

Despite the rapidly accumulating experience with the high volume of teleconsultation adoption in outpatient neurology services during the COVID-19 pandemic, currently, there has not been any scoping review conducted to examine the existing evidence about service process factors from patients and clinicians experiences to our knowledge. Thus, the purpose of this scoping review is to examine existing literature on patients’ and clinicians’ experiences in outpatient neurology teleconsultation during the COVID-19 pandemic to identify key service process factors that impact their experiences. With the ongoing impact of the pandemic, identification of the key service process factors is the first step in gathering new evidence and acquiring new knowledge in service quality during the rapid expansion of teleconsultation, especially since teleconsultation is likely to evolve into common practice in outpatient neurology. In this review, teleconsultation is defined as synchronous consultation between a physician or advanced practice provider and a patient at the patient’s home to provide diagnostic or therapeutic advice through telephone or video conference [31]. This scoping review addresses the research question: “what service process factors of teleconsultation are perceived to have the most impact on patients’ and clinicians’ teleconsultation experiences in outpatient neurology clinics following the rapid shift to teleconsultation during the early stages of COVID-19 outbreak?”

## Methods

The scoping review framework by Arksey & O’Malley served as the framework to structure this review [32]. We applied the five-stage analytic method, which is: (1) identifying the research question, (2) identifying relevant studies, (3) selecting relevant studies, (4) charting the data, and lastly, and (5) collecting, summarizing, and reporting the results [32]. The Preferred Reporting Items for Systematic Reviews and Meta-Analyses (PRISMA) extension for Scoping Reviews guided the reporting of this study [33]. An additional PRISMA checklist shows this in more detail (see Additional file 1).

### Search strategy (identifying relevant studies)

The search strategy for this study was developed by both GM and TT (a research librarian). The search statement comprised both indexed and free-text terms to capture the three main concepts: (1) Virtual consultation, (2) Neurological services, and (3) Service quality assessment. Each of these concepts was captured using the appropriate subject headings (i.e., MeSH and Emtree) along with corresponding natural language keywords that were modified with truncation and phrase search techniques. The final literature search was conducted on April 17, 2021, in four major health sciences databases, including the Cumulative Index to Nursing and Allied Health Literature (CINAHL), APA PsycINFO, PubMed and Scopus.

Since each database has distinctive search functionality and parameters, individually tailored search statements were developed with appropriate search filters. Final search statements were devised through an interactive process to ensure that relevant articles were included while irrelevant ones were excluded. For instance, the initial search contained a large set of articles on “rehabilitation” in neurological services, which is out of scope for this study. As this skewed the search precision, the search statement was revised to exclude this concept. The search was further limited to the English language, peer-reviewed studies that were published from January 2020 to April 2021. The date specification narrowed the search results to articles published since the onset of the COVID-19 pandemic. Final search statements, along with a list of search results, were downloaded from each database for the title and abstract screening.

Some examples of keywords and indexed terms used for this literature search include: (1) virtual Consultation: telemedicine, e-consult, remote consultation, videoconferencing; (2) neurological Services: stroke, neurology, neurosurgical procedures, neurologic examination, neurologists; and (3) service quality assessment: quality assessment, patient satisfaction, quality of health care, and surveys and questionnaires. A detailed major search terms and search strategy, shows this in more detail (see Additional file 1).

### Study selection

The inclusion criteria specified that studies must: (1) be undertaken during the COVID-19 outbreak with a focus on a response to service change due to the pandemic; (2) report on virtual synchronous neurology consultations between a physician or an advanced practice provider and a patient over the age of 18 from a home setting; (3) report on patient and/or physicians’/advanced practice providers’ experience of teleconsultation; and (4) be qualitative, quantitative, or mixed-method peer-reviewed original research. Studies were excluded if the results did not apply to the adult population or the results were not reported separately between synchronous and asynchronous (email, APP, text message, or messaging via web-portal) telemedicine.

**Fig. 1:**
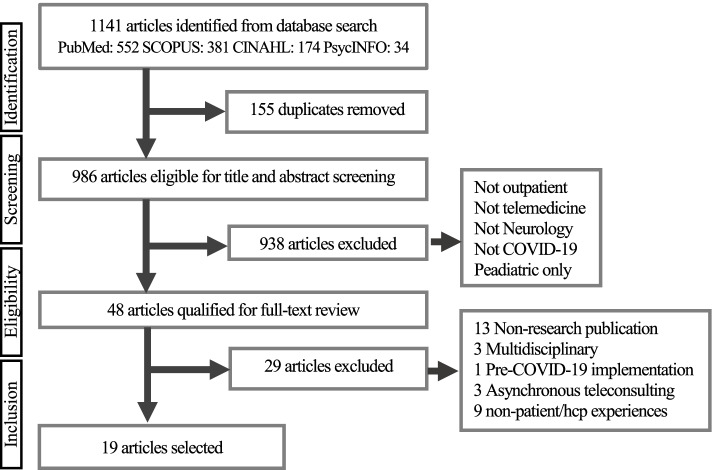
Scoping Review Flow Diagram of Article Selection Process

After the title-abstract screening for relevancy by one reviewer (GM), the second reviewer (TT) randomly reviewed 8% of the title-abstract articles. A google document was created during the screening process. The results were compared, and discrepancies were resolved by making an inclusion or exclusion decision through team discussion. GM and TT reviewed the full-text against inclusion and exclusion criteria. The articles that were chosen for full-text screening were shared with the research team. The level of agreement among the two reviewers was high (98%). The detailed steps of the systematic literature search can be found in the flow chart (Fig. 1). A total of 1141 articles were screened for eligibility. Forty-eight were included for the full-text examination, of which 29 were excluded as they did not meet the eligibility criteria. As a result, 19 articles qualified for this scoping review.

### Charting the data

Thematic analysis was used in this scoping review to identify, analyze, and report patterns (themes) within the data. Braun and Clarke have outlined six steps in undertaking a thematic analysis [34]. A deductive thematic analysis of the results section of selected articles was used to identify service dimensions and their respective service process factors which have the most impact on patients’ and/or clinicians’ teleconsultation service experiences.

### Codebook development

The initial codebook was developed by referencing the telehealth service SERVQUAL questionnaire [29]. An additional table shows this in more detail (see Additional file 1). Some codes were added to reflect the teleconsultation characteristic (e.g., comfort level using technology, human touch), while other codes were combined to avoid redundancy (e.g., voice and image quality were categorized under technological issues). Additional codes (e.g., triage, clinical activities) were identified inductively and added to the codebook during the coding process. There were 21 parent codes for clinicians and 18 for patients in the codebook (see Additional file 1). The codebook modifications were discussed in the biweekly team meetings during the coding process.

### Collecting, summarizing, and reporting the results

We followed Ose’s nine steps to organize all extracted data using Microsoft Excel and Word [35]. For deductive thematic analysis, the results sections of the chosen studies were entered into Microsoft Excel. The paragraphs were broken into sentences, with each sentence representing one code. All relevant data were coded inclusively. The child codes and the quoted texts/sentences were sorted under each parent code in Excel [35]. GM coded the entire dataset. TT independently coded five articles that had 129 codes. The results were compared and reached an initial 83.6% agreement. The discrepancies were discussed, and conflicts were resolved through discussion. Definitions of codes were further clarified where needed.

The frequency of occurrence of each key service process factor was calculated by tallying the total occurrence of each code to identify dimensions and attributes that were most prevalent in the selected studies. Identifying the most frequently mentioned service process factors helped us establish the key determinants or gaps in service quality. We sorted the quotes into sub-themes under each key process factor. We grouped all identified process factors and their themes from both patients’ and clinicians’ experiences. All data generated or analysed during this study are included in this published article (supplementary information Additional file 2).

## Results

A summary of characteristics from the 19 studies is listed in Table 1. The selected publication for this review comprised 13 quantitative cross-sectional survey studies, three cohort studies, two mixed-methods studies, and one qualitative thematic analysis. Study settings included seven general neurology clinics, five epilepsy clinics, two neurosurgery clinics, and five other settings, such as movement disorder, MS or NMOSD, Alzheimer’s, or neurology spine specialties. The majority of studies were primarily conducted in developed countries including the United States (*n* = 8), Germany (*n* = 2), Spain (*n* = 2), United Kingdom (*n* = 1), Ireland (*n* = 1), Norway (*n* = 1), France (*n* = 1), and Italy (*n* = 1). In addition, there were two international survey studies. Single academic institutional authorship was most prevalent (13/19). With regards to the population studied, seven of the articles focused on the clinicians’ perspective, five on the patients’ perspective, and another seven that included both patients’ and clinicians’ perspectives. The participants’ age varied with each study. Nine studies reported the patients’ mean age ranged from 37 to 73.5 years; three reported patients’ mean age above 55 years, only two studies indicated the mean age of clinicians at 41.23 and 42.1. Seventeen studies were carried out from March 17 to July 2020 in the early phase of the COVID-19 global pandemic. Two studies did not specify the study time frame but indicated they were conducted during the COVID-19 pandemic. Regarding the mode of teleconsultation, 12 of the studies looked at both video and telephone, while 4 were telephone only and 3 were video only.

**Table 1:** Descriptive characteristics of the studies (*N* = 19)

#	Authors	Location	Methods	Sample size	Clinician	PT^a^	Mode	Age in years	Setting	Study period
1	Alonso et al. (2021) [16]	South American (4 out of 14 countries)	Cross-sectional study	*N* = 129	Neurologists		Telemedicine with video of 52.3%	mean 41.23	South American MS and NMOSD experts in an outpatient setting	July 3 to 10, 2020
2	Arighi et al. (2021) [36]	Italy	Prospective cohort study	*N* = 108		PT	Video or telephone	mean 73.5	The Alzheimer clinic of a tertiary care academic center	Mid-April to the end of July 2020
3	Casares et al. (2020) [37]	USA	Cross-sectional study	N (PT) =35 N(C) = 5	Physicians	PT	Telephone (12%) Video (88%)	PT mean 37	Epileptic clinic in a tertiary academic center	COVID pandemic
4	Chesnel et al. (2021) [38]	France	Cross-sectional study	N(PT) = 358	Physician	PT	telephone	PT Mean 55.4	A tertiary hospital neuro-urology clinic	March 16 to June 1, 2020
5	Conde-Blanco et al. (2020) [39]	Spain	Cross-sectional study	*N* = 66	Neurologists		Telephone 88%, video 4.5%	35–45 (39.4%) 45–55 (31.8%) > 55 (21.2%)	Spain neurologists in epilepsy clinics	April 14 to May 11, 2020
6	Courtney et al. (2021) [37]	UK	Thematic analysis	*N* = 22	Neurologists and GP with a specialist interest		Telephone or video	20–29 (1/22) 30–39 (6/22) 40–49 (6/22) 50–59 (6/22) 60–69 (3/22)	A tertiary hospital general neurological clinics	June to July 2020
7	Esper et al. (2021) [40]	USA	Retrospective case-control	N(PT) = 686	Clinicians	PT	Video 79.6% Telephone 18.4%	PT mean 64.9	A tertiary academic Movement disorder clinics	March 23 to April 28, 2020
8	Fonseca et al., 2020 [41]	Spain	Cross-sectional study	*N* = 225		PT	telephone	Mean 48.2	A tertiary hospital epilepsy clinic.	March 16 to April 17, 2020
9	Harper et al. (2021) [42]	USA	Cross-sectional study	N(PT) = 1558	Clinicians	PT	video	NA	Tertiary academic neurology Ambulatory Clinics	March 18 to May 8, 2020
10	Kristoffersen et al. (2021) [18]	Norway	Cross-sectional study	*N* = 135	Neurologists		More telephone than video	Mean 42.1	Neurologists in Norway hospital-based outpatient clinics	April 2020
11	Kummer et al. (2021) [43]	USA	Mixed methods	N(PT) = 204 N(C) = 117	Clinicians	PT	video	PT mean 48.8	A tertiary academic Outpatient neurology clinics	April 13 to May 15, 2020.
12	Lovecchio et al. (2021) [44]	International	Cross-sectional study	*N* = 485	Spine surgeons		Telephone 34.6% Video 57.5%	35–44 (68.7%) 45–54 (33.0%)	Members of AO Spine International	May 15 to 31, 20,230
13	McKenna et al. (2020) [45]	Ireland	Cross-sectional study	*N* = 194		PT	Telephone	Mean 47.8	A tertiary hospital General neurology clinics	March 23 to May 25, 2020
14	Mohanty et al. (2020) [46]	USA	Cross-sectional study	N(PT) = 79 N (C) = 40	Neurosurgeons	PT	Video or telephone	NA	A tertiary academic center outpatient neurosurgery clinic	March 1 to July 2, 2021
15	Ryu et al. (2021) [47]	USA	Cross-sectional study	*N* = 14	Neurosurgeons and APPs^c^		Teleconference/ video	NA	A tertiary academic center neurosurgery department	During COVID-19
16	Saliba-Gustafsson et al. (2020) [19]	USA	Mixed methods	*N* = 66	Physicians and APPs		Video visit	NA	A tertiary academic medical center’s ambulatory neurology clinics	March 22 to May 16, 2020
17	Smith et al. (2021) [48]	USA	Cross-sectional study	*N* = 367		PT	Telemedicine Appointment^b^	Non-migraine median age 60 Migraine median age 45	A tertiary Neuroscience outpatient neuro clinics	April 22 to May 18, 2020
18	von Wrede et al. (2020) [49]	Germany	Cross-sectional study	*N* = 239		PT	Telephone (79%) Video (21%)	Mean 41.5	A tertiary Epilepsy center outpatient clinic	March 23 to May 8, 2020
19	Willems et al. (2020) [50]	Germany	Prospective cohort study	*N* = 272	Physicians	PT	Telephone	PT mean 38.7	A tertiary Epilepsy center outpatient clinics	March 17 to May 29, 2020

^a^ *PT* Patients

^b^ The study did not specify the method of telemedicine appointment

^c^*APPS* Advanced practice provider

Most clinicians and patients reported positive experiences in teleconsultation, the definition of satisfaction varied among studies. Between the two populations studied, clinicians had more codes (*n* = 300) than patients (*n* = 101), which is attributed to the fact that there were more qualitative studies examining clinicians’ experiences than of patients. For instance, three (one qualitative and two mixed methods) studies qualitatively examined clinicians’ experiences instead of just one mixed-method study on patients’ experiences. Based on the deductive thematic analysis of the selected studies, we identified six service process factors in outpatient neurology teleconsultation experiences during COVID-19.

### Key service process factors in clinicians’ teleconsultation experiences

There are 300 codes for clinicians’ perceptions of teleconsultation services. The prevalence of codes under each of the SERVQUAL dimensions (tangible, reliability, responsiveness, assurance, and empathy) is listed in Table 2. The most prevalent dimension, process factors, and themes are listed in Table 3. The top six highest-ranking process factors among total coding counts are clinical activities (23%), triage (18.3%), technical issues (14.7%), confidence in care (7.7%), administrative support (6.3%), and communication (5.7%).

**Table 2:** Frequency of each SERVQUAL dimension for clinicians

Dimension	Frequency	%	References from the studies
Tangible comments coded	61	20.3	[37, 39–41, 43, 47, 48, 50]
Reliability comments coded	96	32	[16, 18, 37, 39–41, 43, 46–50]
Responsiveness comments coded	23	7.7	[37, 40, 41, 43, 46–49]
Assurance comments coded	113	37.7	[16, 18, 39–41, 43, 46–51]
Empathy comments coded	7	2.3	[40, 41, 48]
Total SERVQUAL comment coded	300	100	[16, 18, 37, 39–41, 43, 46–51]

**Table 3:** The most frequent SERVQUAL dimensions, process factors, sub-themes among clinicians (*N* = 300)^a^

Dimension	Most frequent process factors	Sub-themes
Assurance (*n* = 113, 37.7%) (The knowledge and courtesy of employees and their ability to inspire trust and confidence)	Clinical activities (*n* = 69): Physical examination (*n* = 32)	The positive role of video in PE Limitation of remote PE Lacking utilization of remote assessment tools Availability of family support
	Confidence in care (*n* = 23)	Video adding confidence Experiences and training adding confidence Perceptions of decreased standard care Unusual conditions (delivery bad news or sensitive information) lowing the confidence
	Communication (*n* = 17)	Perceived risk of misunderstanding Difficult recognizing emotion Difficult establishing trust relationship Superiority of video visits in communication (enhance PE and diagnosis)
Reliability (*n* = 96) (the ability to perform the promised service dependably and accurately)	Appropriate triage (*n* = 55)	Clinical factors: • Follow-up vs New • Screening or stratification • Disease characteristics (severity, stability, acuity, complexity) Patient factors: • Demographic; • Physical or psychological limitation; • Caregiver support; • Access to technology; • Experience in using technology.
	Administrative support (*n* = 19)	Change work flow: Scheduling and registration; Previsit preparation technical, environmental, and medical; Accurate patient information.
Tangible (*n* = 61) (The equipment and personnel)	Technical issues (*n* = 44)	System availability System reliability System connectivity System flexibility Functionality limitation
Responsiveness (*n* = 23) (The willingness to help customers and provide prompt service)	Address patients’ logistical needs (*n* = 13)	Convenience (save time, travel and decrease cost)
Empathy (*n* = 7) (The provision of individual care and attention to customers)	Human touch (*n* = 6)	Losing/missing relationship Lacking empathy: business-like

^a^Original table with selected quotes in Additional file 1

### Key service process factors in patients’ teleconsultation experiences

There are 101 codes for patients’ perceptions of teleconsultation services. The prevalence of codes under each of the SERVQUAL dimensions is listed in Table 4. The most prevalent dimension, process factors, and themes are listed in Table 5. The top six highest-ranking process factors among total coding counts are technical issues (18.8%), logistical needs (16.8%), medical needs (13.9%), communication (11.9%), reliable tests/prescription (7.9%), and home environment (5.9%).

**Table 4:** Frequency of each SERVQUAL dimension for patients

Dimension	Frequency	%	References
Tangible Comments Coded	25	24.8	[36–39, 45–47, 49]
Reliability Comments Coded	15	14.8	[37, 38, 42, 43, 46, 47, 49]
Responsiveness Comments Coded	33	32.7	[19, 37, 38, 42–44, 46, 47]
Assurance Comments Coded	23	22.8	[37, 38, 43, 46, 47, 49]
Empathy Comments Coded	5	5	[38, 47, 49]
Total SERVQUAL Comment Coded	101	100	[19, 36–39, 42–47, 49]

**Table 5:** The most frequent SERVQUAL dimensions, process factors, sub-themes among patients (*N* = 101)^a^

Dimension	Most frequent process factors	Sub-themes
Responsiveness (*n* = 33) (The willingness to help customers and provide prompt service)	Address logistical needs (*n* = 17)	Convenience (saving time, travel and cost)
	Address medical needs (*n* = 14)	Address communicative needs (e.g., understanding care plan, or disease, change medication regiments.)
Tangible (*n* = 25) (The equipment and personnel)	Technical issue (*n* = 19)	Connectivity; Usability; Availability; Family support.
	Home environment (*n* = 6)	Comfort
Assurance (*n* = 23) (The knowledge and courtesy of employees and their ability to inspire trust and confidence)	Communication (*n* = 12)	Situational effectiveness
	Diagnosis (*n* = 5)	Delay and uncertain
Reliability (*n* = 15) (the ability to perform the promised service dependably and accurately)	Tests, prescriptions, Treatments (*n* = 8)	Delay
Empathy (*n* = 5) (The provision of individual care and attention to customers)	Personal attention (*n* = 3)	Present Embarrassing

^a^Original table with selected quotes in Additional file 1

### Six key common service process factors and themes among clinicians and patients

The top six factors that influenced both patients’ and clinicians’ perceptions of teleconsultation experiences are: (1) technical issues, (2) triage, (3) logistical needs, (4) administrative support, (5) clinical activities, and (6) communication. Table 6 shows the six identified key service process factors and themes under each factor.

**Table 6:** The six identified key service process factors and themes among clinicians and patients

Key factors	Themes	Subthemes
Technical issues	System and organizational level Individual level	Availability Connectivity Functionality Flexibility Reliability Technical support Technological capacity
Triage	Patients’ preference Clinical appropriateness	Patients’ ability to use technology • Demographic • Physical and cognitive impairment • Family support Clinical factors • Disease types • Stability/acuity • Need physical examination
Logistical needs	Convenience	Save time, travel, cost
Administrative support	Virtual workflow issues	Scheduling and registration Pre-visits preparation: technical, environmental and medical Accurate patient information
Clinical activity	Clinicians’ lack of confidence, virtual care experiences and competency	Lack of non-verbal communication • Misunderstanding • Difficult recognizing emotion • Difficult establishing trust relationship Superiority of video • Enable virtual assessment; • Enhance confidence in diagnosis.

The first key service process factor, technical issues, was raised mainly by clinicians who reported significant gaps in this area, especially regarding functionality limitations, reliability (connectivity issues), availability, accessibility, system flexibility (rigid schedule), lack of technical support and training [19, 36, 37, 39, 42, 43, 47, 51]. For instance, clinicians indicated that patients’ technological capacity was a barrier to successful teleconsultation [19, 47]. Although one study reported that none of their patients (mean age 37) expressed internet connectivity issues as barriers [37], according to another one, 31.5% of patients with cognitive impairment (mean age 75.7) failed video consultation mainly due to difficulty in establishing a connection (76.4%) [36]. One study which compared the video to telephone consultation groups reported that only age revealed a statistically significant shift towards a preference to telephone consultation [40].

The second key service process factor, triage, has evolved from a clinical process to a complex process weighing the interest of both patients and clinical standpoints. The patient component includes demographic factors such as age, gender, racial/ethnic minority, social-economic status, psychological or psychological challenges, caregiver support, language barriers, access to technology, and experience with technology [40, 47, 49, 51]. Whereas the clinical component includes disease stability, disease acuity, disease complexity, disease types, new versus follow-up visits, the role of physical examination in decision making, high-risk procedure, and the delivery of bad/sensitive news [18, 19, 37, 39, 42–44, 46, 47, 50, 51]. The clinicians identified that most appropriate patients for teleconsultation were: (1) follow-up patients with well-established diagnosis and requiring regular monitoring, (2) patients with chronic, stable, and uncomplicated conditions, (3) patients being stratified or screened to assess the need for in-person visits, (4) certain conditions or diseases such as headache or epilepsy, (5) older patients or vulnerable population who are unable to attend in-person visits [18, 19, 37, 39, 42–44, 46, 47, 50, 51]. Clinicians identified the most inappropriate patients for teleconsultation were: (1) new patients, (2) those with acute conditions or declined health or physical changes, (3) individuals with life-threatening diagnosis or high-risk treatments, (4) patients needing/requiring hands-on physical examination, (5) individuals with certain types of diseases such as movement disorder or MS; (6) patients with hearing or visual or cognitive impairment; (7) those with language barriers; (8) patients lacking family support; (9) and individuals with low technical capability such as older or low-income patients [18, 19, 37, 39, 42–44, 46, 47, 50, 51]. In terms of the suitability of teleconsultation the older and vulnerable populations, it was considered both appropriate and inappropriate.

The third key service process factor, meeting patients’ logistical needs, plays a crucial role in contributing to a positive teleconsultation experience from both patients’ and clinicians’ perspectives [40, 45, 46, 48, 51]. According to the findings from one publication, 88% of patients agreed that teleconsultation was more convenient than an in-person visit [46]. Other research findings also indicate that the benefits of teleconsultation to patients included reduced unnecessary travel and increased access to healthcare, especially for vulnerable populations, including those with disabilities or lack access to transportation [19, 37, 47].

The fourth key service process factor, administrative support, is an essential component to ensuring the success of teleconsultation [19, 37, 43, 51]. Clinicians identified that lack of administrative support negatively affected their perceptions of teleconsultation [51]. Administrative support is vital to a successful teleconsultation. Many of the studies indicated that clinicians emphasized the need for environmental (e.g., adequate space, optimal camera position, and lighting), technological (e.g., technology availability, access to a virtual platform, working camera and speaker, patients’ technological knowledge and experience assessment, and opportunity for a trial run before teleconsultations), and clinical preparations (e.g., medication reconciliation, investigation results, and past medical history) with direct patient and family involvement [19, 36, 37, 51]. For instance, having family support from the younger generation has significantly increased the success of video consultations among the elderly with cognitive impairment [36]. These studies reported that clinicians experienced difficulty connecting to patients, such as when patients were unavailable at the time of appointment or were engaged in other activities (working or driving), or the patients were simply not prepared for teleconsultation [19, 37]. Kummer et al. found that 8.3% of video consultations had to switch to different modality due to lack of technical preparation [43].

The fifth key service process factor, clinical activities, is a crucial factor identified in eleven of the studies. The concerns of clinical activities include clinicians’ concerns with their inability to perform clinical activities, primarily physical examinations during a teleconsultation visit [16, 18, 19, 36, 38, 39, 43, 44, 46, 47, 51]. Some studies uncovered that part of the obstacles to clinical activities with teleconsultation is due to clinicians’ lack of utilization of existing electronic resources and remote assessment tools [16, 39]. One study found that even though hospitals subscribe to electronic medical record (EMR), 30.3% of clinicians still reported being unable to access the EMR, and almost 20% of clinicians could not make electronic prescriptions [39]. Correspondingly, other studies have shown that increased experience and training with virtual care correlated with improvement in clinicians’ ability to diagnose and develop treatment plans virtually and boost their acceptability and satisfaction with teleconsultation [43, 44].

Lastly, the sixth key service process factor, communication, dealt primarily with the perceived quality of communication between audio-only and video modes of teleconsultation. For instance, clinicians disclosed that the difficulty with audio-only teleconsultation was a barrier to holistically obtaining information, which in turn also negatively affected the patient-physician relationship. The lack of visual cues interfered with the clinicians’ ability to interact with their patients, as they could not access non-verbal communication. This was especially problematic for physicians with patients with hearing or cognitive impairments and those with language barriers [19, 38, 51]. From the clinicians’ perspective, teleconsultation using video platforms had added value by giving them access to non-verbal communication allowing them to visually and verbally assess their patients’ responses and reactions. In turn, video, as opposed to audio-only, teleconsultations enabled physicians to diagnose with more confidence, resulting in physicians expressing a more successful experience [44, 47, 51]. However, with technical issues and a lack of administrative support, this is an unmet need for clinicians to use video to optimize their teleconsultation experiences.

## Discussion

Without a doubt, the COVID-19 pandemic has been a catalyst for teleconsultations’ rapid expansion in many health sectors. The impact of the pandemic, which forcibly halted in-person services in most sectors globally, sparked the rapid and massive adaptation of virtual communication due to social distancing restrictions [52]. According to one study, teleconsultation requests in outpatient neurology were significantly associated with the subjectively perceived threat by SARS-CoV-2 (*p* = 0.004) [50]. Since the start of the COVID-19 pandemic, teleconsultation has become an essential tool in outpatient service delivery [41]. The rapid expansion of teleconsultation in outpatient neurology service has allowed us to gain new insight into service quality as the scope of adaptation has never been seen before in healthcare history.

This scoping review identified six key service process factors that affected the teleconsultation experiences at outpatient neurology services from both patients’ and clinicians’ perspectives. While four of the identified service factors, technical issues, logistical needs (convenience), communication, and ability to perform clinical activities, were consistent with findings from the pre-COVID era, the remaining two factors, appropriate triage and administrative support are new findings from this review. Our review has highlighted that appropriate triage is essential for a successful teleconsultation, especially considering patients’ technological capacity, preference (logistical needs), disease characteristics, and the ability of their clinician to perform a physical examination for diagnosis and formulating a treatment plan. In addition, this review also determined that appropriate administrative support is essential to a successful teleconsultation visit by equipping both patients (by assessing patients’ technological capacity, assisting technical issues, and supporting patients/caregivers) and clinicians (by providing well-prepared documents, accurate patient information, vital signs, and medication reconciliation) with the necessary tools, support, and information. Therefore, the findings from this review will be essential to ensuring a high-quality teleconsultation visit in neurology outpatient.

### Exacerbated technical issues during COVID-19 for vulnerable population

Before the COVID-19 outbreak, there were not as many technical issues reported in outpatient neurology teleconsultation when done at a satellite clinic [11, 13, 53–55]. According to one study, veterans with chronic neurological diseases who had follow-up teleconsultations at satellite clinics rarely encountered technical problems [53]. Additionally, according to another study, there were few same-day cancellations (2/64) in follow-up teleconsultation for rare neurological diseases due to technical issues [11]. However, the amount and extent of the technical issue encountered became prominent in follow-up visits with teleconsultation from a home setting. Teleconsultations with patients at home were manifested with technical troubles and having to do with patients’ discomfort with technology, which often necessitated assistance from younger caregivers [10, 56].

From a technological perspective, although the COVID-19 pandemic has significantly increased the use of digital technologies in nearly every aspect of our lives, it has also deepened digital inequity [57]. Digital inequality exasperated by the rapid, large-scale adaptation in telecommunications has proven to be a significant barrier to the vulnerable patient population [36]. Due to social distancing, much of the teleconsultations since the COVID-19 outbreak were conducted from the patients’ homes, rather than a satellite clinic. Without proper assistance and experience with telecommunication has gravely contributed to the technical difficulties encountered at the patients’ end. Our review has confirmed that access to appropriate technology, patients’ digital literacy, language, physical or cognitive capability, coupled with the medical needs of the elderly and vulnerable population, have significantly limited access to teleconsultation [19, 36, 37, 47].

According to a cross-sectional population study based on data collected in 2018 of community-dwelling adults over the age of 65, 38% of all older adults in the United States were not ready for video visits, mainly because of inexperience with technology. In addition, telephone visits would be problematic for 20% of this population due to having hearing impairments, difficulty communicating or suffering from dementia [58]. A literature-based framework explored the four key age-related barriers influencing mobile health usability, enabling further evaluation of teleconsultation in the geriatric population [59]. Digital health literacy has become a new social determinant of health [56]. As such, healthcare policymakers must consider technology-enabled services to counter the effects of this determinant [56]. Both political and community interventions must be enacted to ensure that appropriate supports are in place and to mitigate the adverse effects of the pandemic and the social health inequalities [57].

### Clinical activities: clinicians’ moral distress and the role of physical examination

The limitation of the remote physical examination has been a significant concern in outpatient neurology teleconsultation before the COVID-19 pandemic. This was likely the primary reason that majority of teleconsultations were done only for follow-up patients after the initial in-person assessment. In fact, prior to the outbreak, teleconsultation was positioned as an optimal solution for remote longitudinal care as a physical examination is not as vital for follow-up patients [60]. Studies examining new but non-urgent neurology patients assessed via teleconsultation conducted in satellite clinics with the aid of a professional telepresenter, demonstrated the noninferiority of virtual consultations for diagnosis, especially given the high level of patient acceptance [12, 13]. In fact, the assistance of a professional telepresenter could highly enhance the accuracy of remote physical examinations by ensuring that any vital signs and symptoms that are relevant to diagnosis are not overlooked [60]. A 2019 review of telemedicine in neurology by the American Academy of Neurology established that diagnosis in traumatic brain injury, dementia, Parkinson’s disease (PD), and MS, via teleconsultation, can be as effective as in person [5]. However, this study had several limitations. For instance, the analysis did not distinguish between studies that evaluated inpatient versus outpatient groups [5]. Moreover, some of the studies included were performed in artificial settings, involved the aid of a telepresenter, had a small sample size, or only comprised of the stable, unchanged non-acute patient population [5].

Since the COVID-19 outbreak, teleconsultation has been widely utilized with new and follow-up patients in a home setting without the luxury of a professional telepresenter to assist with the technology or the examination. The rapid adoption of teleconsultation in neurology has compelled many clinicians to provide care without appropriate training or credentialing to use this unique service delivery model effectively. Performing remote physical examinations without providing patients appropriate assistance and clinicians the needed training could gravely affect diagnosis and treatment plan. Our review confirms that the constraints of conducting a physical examination virtually has often been translated into a sense of diminished confidence in service quality for the clinician [10]. The impact of the COVID-19 outbreak on health care has immensely altered the standard practice model, compelling clinicians to compromise on the widely accepted care standards to reduce the impact of the highly infectious and virulent disease. The lack of standard best practice guidelines for teleconsultation among neurology sub-specialties has pressured the ethical and moral responsibility of providing good quality of care directly in the hands of individual clinicians. According to Courtney et al., clinicians’ heightened awareness of the risks associated with diagnostic uncertainty led to much of the reluctance with virtual examination resulting in ‘unknown unknowns’ [51]. Therefore, we recommend further research investigating clinicians’ moral distress in teleconsultation during COVID-19.

Despite the explosion of teleconsultation in neurology, some neurology specialties still have yet to adopt physical examination into a digital landscape [36, 44, 47]. For example, Casares et al. found that providers preferred in-person appointments for complex cases in a follow-up epilepsy clinic during the COVID-19 pandemic, even when the visits rely mainly on the interview rather than the physical examination [43]. The limitations with adopting traditional neurological examinations into a teleconsultation model could be addressed with innovations in digital health and the use of remote monitoring devices [43, 47]. With a vulnerable patient population, having family members assist clinicians with remote physical examinations has proven vital in ensuring patients’ safety [19]. Therefore, it would be beneficial to conduct further research on the reliability and safety of family-supported remote physical examinations in undiagnosed patients. Lastly, further research identifying the components of the in-person examination that are essential for the clinical decision-making process needs to be deciphered to meet documentation requirements [43, 46].

### Communication: more negative perceived by clinicians

Before the COVID-19 pandemic, patients’ satisfaction with the quality of communication during teleconsultation was high but mostly among follow-up patients or in outpatient neurology satellite clinics with the assistance of a telepresenter [13, 53–55, 61]. Contrarily, some follow-up patients at home-setting expressed discomfort with telecommunication and indicated a preference for in-person interaction as they experienced greater ease communicating and found the physical interaction more reassuring and personal [10, 56]. Unfortunately, clinicians’ satisfaction with teleconsultation communication quality was less examined in outpatient neurology settings.

Since the COVID-19 outbreak, teleconsultation visits have been mainly conducted with patients from a home setting. Many of these teleconsultations have been with new patients who have had no established relationship with the clinician, which may have contributed negatively to their perceived quality of communication. Interestingly, teleconsultation studies show that patients had more positive perceptions than clinicians. Four studies that used telephone and video modalities indicated that most patients felt communication was effective and sufficient with teleconsultations [37, 46, 49, 50]. Contrarily, clinicians expressed more negative experiences towards communication in five of the studies that used both telephone and video modalities, especially regarding concerns about decreased personal connections and risk of misunderstanding [19, 39, 47, 51]. Further research is needed to explore patient-clinician relationships in a virtual setting in terms of the role of verbal and non-verbal communication from both clinicians’ and patients’ perspectives. Non-verbal communication enables the clinician to observe patients’ physical appearance, eye contact, or emotions and assess the home environment, providing more information in formulating diagnosis and treatment plans [19, 62]. The added value of non-verbal communication on patient-clinician relationships and the ability to perform clinical activities may differ among specialties and diverse patient populations, requiring further exploration.

### Meeting logistical needs (convenience): a contributing factor

A systematic review of telehealth services pre-COVID-19 concluded that convenience (travel-saving, time-saving, and cost-saving) is one of the most significant factors influencing patients’ satisfaction [63]. The outpatient neurology teleconsultation is no exception. Convenience by meeting patients’ logistical needs (travel, transportation, missing work and finical constrain) is one factor that influences patients’ positive perceptions of the personal benefits of teleconsultation [5, 8–10, 54, 61, 64]. Interestingly, the distance was not statistically associated with patient satisfaction in outpatient neurology teleconsultation [11, 61]. Another study that examined outpatient neurological teleconsultation follow-up visits found that 30% of local patients chose teleconsultation, which indicated that patients might benefit for a variety of reasons other than distance [9].

With COVID-19 restrictions, teleconsultation is undoubtedly preferable to the alternative, not receiving any care [42]. Our review confirmed that both patients and clinicians appreciated the convenience of teleconsultation as a factor swaying their positive perceptions of the teleconsultation service quality. However, convenience does not equate to quality of care. Therefore, although convenience is an important factor, understandably, preference for it could easily influence patients’ evaluation of teleconsultation service regarding the quality of care [10].

### A improved triage process: finding the middle ground

A new insight revealed in this review is that teleconsultation triage has become a complex collaborative process involving both patients and clinicians. Patient selection for teleconsultation requires careful consideration to optimize care and respect preferences from both patients’ and clinicians’ points of view [19]. The triage process needs to be established by considering patients’ technological competency, their preference, disease characteristics, and the role of physical examination in the diagnosis and formulation of a treatment plan [18, 19, 37, 39, 42–44, 46, 47, 50, 51].

Prior to the outbreak, teleconsultation in outpatient neurology was mainly limited to follow-up patients with a confirmed diagnosis. With this mentality, many clinicians tend to regard teleconsultation as unsuitable for new referrals or follow-up patients with worsening symptoms [19, 37, 42, 51]. Interestingly, in contrast, one of the studies observed that the utilization of teleconsultation was high for both new and returning patients. However, it could have been due to underlying fears of contracting COVID-19 confounding this observation [19]. Clinicians believe that medical conditions that depend on medical history-guided diagnostic decision-making are more appropriate than those that are neurological examination-guided [18, 45, 46]. Certain conditions (e.g., headache and epilepsy) were perceived as more suitable for teleconsultation than others (e.g., MS, movement disorders, or myelopathy) [18, 44, 48]. As a result, a disease-specific triage algorithm is necessary to guide patient selection.

Some clinicians expressed that teleconsultation might empower their patients with management options, leading to an excessive number of consultations in an already overused and high-demand specialty [47]. Clinicians also expressed concerns that patients may find teleconsultation too convenient and opt-out of recommended in-person visits [19]. Thus, they were apprehensive about patients preferring the convenience of teleconsultation against their clinical recommendation for an in-person visit. This finding has not been reported in studies before the COVID-19 pandemic. The possible reasons could be that the COVID-19 restrictions compelled many new patient intakes via teleconsultation prior to developing clear patient selection criteria or virtual care guidelines.

### Administrative support: a new virtual care workflow

Another new insight uncovered from this review is that lack of proper administrative support negatively affected clinicians’ perceptions of teleconsultation [19, 37, 43, 51]. The lack of protocols prior to the teleconsultation, specifically with regards to technology set up, check-in processes, procedures with vital sign assessment, and medication reconciliation, reflect a need to establish a new administrative virtual care workflow [19, 37, 43, 51]. Unfortunately, teleconsultation, compared to an in-person visit, seems to have generated more work for clinicians and administrative staff. This, in turn, is affecting workflow efficiency and widening the gap between the needs of a successful teleconsultation and the actual administrative support available [19, 43, 51]. The rapid adaptation of teleconsultation since the onset of the COVID-19 pandemic, without the appropriate organizational planning and support, in addition to the strains of staffing deployment due to COVID-related care as well as keeping pace with transitioning workflow between telephone, video, and in-person visits, may have contributed to the maladaptation [43]. The onus of establishing new administrative protocols to manage virtual workflow rests at the organizational level rather than with the individual clinicians. Keeping in mind that technology is a “tool”, not the “solution.”, it necessitates building a sustainable administrative virtual workflow model to support the frontline clinicians [65].

### Future teleconsultation service model in outpatient neurology

Our review has highlighted six key service process factors that must be addressed to improve teleconsultation service quality. Two models of care could address some of the issues highlighted in the six key service process factors identified. On the one hand, a hybrid model or a multimodal system that is comprised of both virtual and in-person visits would help mitigate some of the barriers faced by vulnerable patient populations, such as those who have disabilities or issues accessing transportation [47]. The added value of teleconsultation affords new opportunities to collaborate, incorporate family support, and ensure continuity of care [18, 19, 38]. On the other hand, a disease-specific model would address the diverse needs of the various neurology subspecialty groups. For instance, while some subspecialties, such as oncology neurosurgery, could accommodate follow-up patient intake with teleconsultation, others, such as functional neurosurgery, may be stringent with follow-up visits to be done in-person [46]. As such, further research is needed to identify the types of disease and the needs of varying patient populations to ensure that appropriate care is delivered using best practices to accommodate both clinicians’ and patients’ provisions.

### Strengths and limitations

There are two strengths of this review. First, we strictly applied the systematic scoping review framework. Second, we applied the SERVQUAL model as a theoretical framework to classify the factors that impact the perceptions of teleconsultation. This review focuses on experiences of teleconsultation during the COVID-19 outbreak based on qualitative, quantitative, or mixed-method peer-reviewed original research published from January 2020 to April 2021. Due to the restrictions imposed during the COVID-19 pandemic, many of the well-established protocols and standards of practices relating to privacy, security, reimbursement, and appropriate credentialing in the pre-COVID era were relaxed [3, 18]. The teleconsultations conducted with the onset of the COVID-19 pandemic have vastly broadened the width and depth of teleconsultation adoption in both urban and remote areas, with new and follow-up patients accessing care from a home setting, who have or have yet to be diagnosed. Our review has contributed to gaining a better understanding of outpatient teleconsultation service quality at-home settings.

Our scoping review has many limitations. First, due to the nature of a scoping review, it is challenging to interpret patients’ or clinicians’ experiences when the little context was provided during the coding process. Second, the selected studies were conducted in broad geographic areas, across many neurology specialties, with varying methodologies. The heterogeneous nature of the selected studies made it challenging to identify specific factors in a particular group of the patient population. However, in line with the advantages of a scoping review methodology, is that it offers a broader lens as it allows for analysis of a variety of study designs and patient populations in mapping the unfamiliar phenomenon [32].

## Conclusion

Our scoping review identified six key service process factors of teleconsultation that had the most impact on patients’ and clinicians’ teleconsultation experiences during the COVID-19 outbreak. Compared to the pre-COVID outpatient neurology teleconsultation literature, we identified two new findings: the need to develop and implement a new triage system model and define gaps in an administrative workflow to incorporate virtual care. These findings will help inform a best practice model by guiding researchers, clinicians, and policymakers to design theory-informed teleconsultation services tailored to the needs of neurology patients and clinicians. Thus, these findings lay the groundwork to improve teleconsultation implementation in outpatient neurology services.

## Supplementary Information

**Additional file 1: Appendix A.** Items in SERVQUAL model presented by Zeithaml et al.(1990). **Appendix B.** Preferred Reporting Items for Systematic reviews and Meta-Analyses extension for Scoping Reviews (PRISMA-ScR) Checklist. **Appendix** ***C.*** *Major* search terms statements April 17, 2021. Appendix D. The SERVQUAL questionnaire of a telehealth program in the case hospital conducted by Yin et al. (2016) **Appendix D.** The SERVQUAL questionnaire of a telehealth program in the case hospital conducted by Yin et al. (2016). **Appendix E.** SERVQUAL model codebook. **Appendix F.** The most frequent SERVQUAL dimensions, process factors, sub-themes with selected quotes. **Appendix G.** The most frequent SERVQUAL dimensions, process factors, sub-themes and selected quotes among patients.

**Additional file 2.** Raw dataset.

## Data Availability

The list of articles.

## Abbreviations

OTN: Ontario’s Telemedicine Network
COVID-19: Corona virus disease 2019
KHSC: Kingston Health Science center
MS: Multiple sclerosis
NMOSD: Neuromyelitis optica spectrum disorders
SERVQUAL: Service quality model
PRISMA: The Preferred Reporting Items for Systematic Reviews and Meta-Analyses
SARS-CoV-2: Severe acute respiratory syndrome coronavirus 2
PD: Pakinson’s Disease
EMR: Electronic medical record
