# Supplementary material for: Service process factors affecting patients’ and clinicians’ experiences on rapid teleconsultation implementation in out-patient neurology services during COVID-19 pandemic: a scoping review
Source: BMC Health Serv Res. 2022 Apr 22;22:534. doi: 10.1186/s12913-022-07908-4 (PMC9026006; doi:10.1186/s12913-022-07908-4)
Supplement: Supplementary file 1 — Additional file 1: Appendix A. Items in SERVQUAL model presented by Zeithaml et al.(1990). Appendix B. Preferred Reporting Items for Systematic reviews and Meta-Analyses extension for Scoping Reviews (PRISMA-ScR) Checklist. Appendix C. Major search terms statements April 17, 2021. Appendix D. The SERVQUAL questionnaire of a telehealth program in the case hospital conducted by Yin et al. (2016) Appendix D. The SERVQUAL questionnaire of a telehealth program in the case hospital conducted by Yin et al. (2016). Appendix E. SERVQUAL model codebook. Appendix F. The most frequent SERVQUAL dimensions, process factors, sub-themes with selected quotes. Appendix G. The most frequent SERVQUAL dimensions, process factors, sub-themes and selected quotes among patients. [file 12913_2022_7908_MOESM1_ESM.docx]

**Additional file 1**

**Appendix A**. Items in SERVQUAL model presented by Zeithaml et al (1990)

| Dimension description | Item description |
| --- | --- |
| **Tangible:**  facilities, equipment, and the presence of personnel | 1. Up-to-date equipment  2. Visually appealing physical facilities  3. Neat-appearing employees  4. Visually appealing materials associated with the service |
| **Reliability:**  ability to perform the promised service responsibly and accurately | 5. The company keeps its promises to do something by a certain time  6. The company shows a sincere interest in solving the customer’s problem  7. The company performs the service right the first time  8. The company provides its services at the time it promises to do so  9. The company insists on error-free records |
| **Responsiveness**:  willingness to provide help and a prompt service to customers | 10. Employees of the company tell customers exactly when services will be performed  11. Employees of the company give prompt service to customers  12. Employees of the company are always willing to help customers  13. Employees of the company are never too busy to respond to customer |
| **Reassurance**:  the knowledge and courtesy of employees and their ability to inspire trust and confidence | 14. The behaviour of employees of the company instills confidence in customers  15. Customers of the company feel safe in their transactions  16. Employees of the company are consistently courteous with customers  17. Employees of the company have the knowledge to answer customer’s questions |
| **Empathy**  caring and understanding, which a company provides and/or offers its customers in terms of its individualized and personalized attention | 18. The company gives customers individual attention  19. The company has operating hours convenient to all its customers  20. Employees of the company give customers personal attention  21. The company has the customer’s best interests at heart  22. The employees of the company understand the specific needs of their |

Sources: adapted from Zeitham et al. (1990).

**Appendix B.** Preferred Reporting Items for Systematic reviews and Meta-Analyses extension for Scoping Reviews (PRISMA-ScR) Checklist

| **SECTION** | **ITEM** | **PRISMA-ScR CHECKLIST ITEM** | **REPORTED ON PAGE #** |
| --- | --- | --- | --- |
| **TITLE** | | | |
| Title | 1 | Identify the report as a scoping review. | 1 |
| **ABSTRACT** | | | |
| Structured summary | 2 | Provide a structured summary that includes (as applicable): background, objectives, eligibility criteria, sources of evidence, charting methods, results, and conclusions that relate to the review questions and objectives. | 1-2 |
| **INTRODUCTION** | | | |
| Rationale | 3 | Describe the rationale for the review in the context of what is already known. Explain why the review questions/objectives lend themselves to a scoping review approach. | 3-5 |
| Objectives | 4 | Provide an explicit statement of the questions and objectives being addressed with reference to their key elements (e.g., population or participants, concepts, and context) or other relevant key elements used to conceptualize the review questions and/or objectives. | 6 |
| **METHODS** | | | |
| Protocol and registration | 5 | Indicate whether a review protocol exists; state if and where it can be accessed (e.g., a Web address); and if available, provide registration information, including the registration number. | na |
| Eligibility criteria | 6 | Specify characteristics of the sources of evidence used as eligibility criteria (e.g., years considered, language, and publication status), and provide a rationale. | 7 |
| Information sources* | 7 | Describe all information sources in the search (e.g., databases with dates of coverage and contact with authors to identify additional sources), as well as the date the most recent search was executed. | 7-8 |
| Search | 8 | Present the full electronic search strategy for at least 1 database, including any limits used, such that it could be repeated. | 8 |
| Selection of sources of evidence† | 9 | State the process for selecting sources of evidence (i.e., screening and eligibility) included in the scoping review. | 8-9 |
| Data charting process‡ | 10 | Describe the methods of charting data from the included sources of evidence (e.g., calibrated forms or forms that have been tested by the team before their use, and whether data charting was done independently or in duplicate) and any processes for obtaining and confirming data from investigators. | 9 |
| Data items | 11 | List and define all variables for which data were sought and any assumptions and simplifications made. | 9-10 |
| Critical appraisal of individual sources of evidence§ | 12 | If done, provide a rationale for conducting a critical appraisal of included sources of evidence; describe the methods used and how this information was used in any data synthesis (if appropriate). | na |
| Synthesis of results | 13 | Describe the methods of handling and summarizing the data that were charted. | 9-10 |
| **RESULTS** | | | |
| Selection of sources of evidence | 14 | Give numbers of sources of evidence screened, assessed for eligibility, and included in the review, with reasons for exclusions at each stage, ideally using a flow diagram. | 10-13 |
| Characteristics of sources of evidence | 15 | For each source of evidence, present characteristics for which data were charted and provide the citations. | 11-13 |
| Critical appraisal within sources of evidence | 16 | If done, present data on critical appraisal of included sources of evidence (see item 12). | na |
| Results of individual sources of evidence | 17 | For each included source of evidence, present the relevant data that were charted that relate to the review questions and objectives. | 13-16 |
| Synthesis of results | 18 | Summarize and/or present the charting results as they relate to the review questions and objectives. | 16-20 |
| **DISCUSSION** | | | |
| Summary of evidence | 19 | Summarize the main results (including an overview of concepts, themes, and types of evidence available), link to the review questions and objectives, and consider the relevance to key groups. | 20-30 |
| Limitations | 20 | Discuss the limitations of the scoping review process. | 30 |
| Conclusions | 21 | Provide a general interpretation of the results with respect to the review questions and objectives, as well as potential implications and/or next steps. | 31 |
| **FUNDING** | | | |
| Funding | 22 | Describe sources of funding for the included sources of evidence, as well as sources of funding for the scoping review. Describe the role of the funders of the scoping review. | na |

**Appendix C.** Major search terms statements April 17, 2021

## Virtual Consultation

### Indexed Terms:

- Ambulatory Care (MH PsycINFO, CINAHL MW, Scopus INDEXTERMS, MeSH)
- Ambulatory Care Facilities (MH PsycINFO, MeSH)
- Distance Counseling (MH PsycINFO, CINAHL MW, Scopus INDEXTERMS, MeSH)
- Home Care Services (MH PsycINFO, CINAHL MW, Scopus INDEXTERMS, MeSH)
- Outpatients (MH PsycINFO, CINAHL MW, Scopus INDEXTERMS, MeSH)
- Remote Consultation (MH PsycINFO, CINAHL MW, Scopus INDEXTERMS, MeSH)
- Telehealth (CINAHL MW, Scopus INDEXTERMS)
- Telemedicine (MH PsycINFO, CINAHL MW, Scopus INDEXTERMS, MeSH)
- Teleneurology (CINAHL MW, Scopus INDEXTERMS)
- Telenursing (CINAHL MW, Scopus INDEXTERMS)
- Telestroke (Scopus INDEXTERMS, CINAHL MW)
- Videoconferencing (MH PsycINFO, CINAHL MW, Scopus INDEXTERMS, MeSH)

### Free-text Terms:

- Digital health
- eConsult
- eHealth
- electronic visit
- eVisit
- outpatient
- Remote care
- Remote consult
- Remote health
- Teleconsult
- Telehealth
- Telemedicine
- Teleneurology
- Telestroke
- Televisit
- Video Visit
- Virtual care
- Virtual health

## Patient/Clinician Perspective

### Indexed Terms:

- Attitude of Health Personnel (MH PsycINFO, CINAHL MW, Scopus INDEXTERMS, MeSH)
- Attitude to Health (MH PsycINFO, CINAHL MW, Scopus INDEXTERMS, MeSH)
- Job Satisfaction (MH PsycINFO, CINAHL MW, Scopus INDEXTERMS, MeSH)
- Nurse Patient Relations (MH PsycINFO, CINAHL MW, Scopus INDEXTERMS, MeSH)
- Outcome Assessment Health Care (MH PsycINFO, CINAHL MW, Scopus INDEXTERMS, MeSH)
- Outpatients (MH PsycINFO)
- Patient Preference (CINAHL MW, Scopus INDEXTERMS)
- Patient satisfaction (MH PsycINFO, CINAHL MW, Scopus INDEXTERMS, MeSH)
- Patient-Centered Care (MH PsycINFO, CINAHL MW, Scopus INDEXTERMS, MeSH)
- Personal satisfaction (MH PsycINFO, CINAHL MW, Scopus INDEXTERMS, MeSH)
- Physician Patient Relations (MH PsycINFO, CINAHL MW, Scopus INDEXTERMS, MeSH)
- Practice Patterns Nurses (MH PsycINFO, CINAHL MW, Scopus INDEXTERMS, MeSH)
- Practice Patterns Physicians (MH PsycINFO, CINAHL MW, Scopus INDEXTERMS, MeSH)
- Professional patient Relations (MH PsycINFO, MeSH)
- Professional-Client Relations (Scopus INDEXTERMS, CINAHL MW)
- Professional-Patient Relations (CINAHL MW, Scopus INDEXTERMS)
- Quality of Health Care (MH PsycINFO, CINAHL MW, Scopus INDEXTERMS, MeSH)
- Surveys and Questionnaires (MH PsycINFO, CINAHL MW, Scopus INDEXTERMS, MeSH)

### Free-Text Terms:

- Patient-centered
- User-centered
- Combination of: (Patient, Health Personnel, Provider, Physician, Nurse, Neurologist, Clinician, User, Outpatient, Ambulatory) AND (satisfaction, experience, perspective, Evaluation, Survey, Lessons, Practices, Observation, Attitude OR View)

## Neurology

### Indexed Terms:

- Cerebrovascular Disorders (MH PsycINFO, CINAHL MW, Scopus INDEXTERMS, MeSH)
- Neurologic Examination (MH PsycINFO, CINAHL MW, Scopus INDEXTERMS, MeSH)
- Neurologists (MH PsycINFO, CINAHL MW, Scopus INDEXTERMS, MeSH)
- Neurology (MH PsycINFO, CINAHL MW, Scopus INDEXTERMS, MeSH)
- Neurosurgical Procedures (MH PsycINFO, CINAHL MW, Scopus INDEXTERMS, MeSH)
- Stroke (MH PsycINFO, CINAHL MW, Scopus INDEXTERMS, MeSH)
- Stroke Units (CINAHL MW, Stroke Units (Scopus INDEXTERMS)

### Free-Text Terms:

- Stroke
- Neurology
- Neurologist

## Full Search Statements for all 4 Databases

### Pubmed 552 (April 17, 2021)

(("2019/11/01"[Date - Publication] : "3000"[Date - Publication])) AND (((((telemedicine[MeSH] OR videoconferencing[MeSH] OR remote consultation[MeSH] OR Ambulatory Care Facilities[MeSH] OR Outpatients[MeSH] OR Ambulatory Care[MeSH] OR Distance Counseling[MeSH] OR Home Care Services[MeSH] OR Tele?medicine[TiAb] OR Tele?health*[TiAb] OR Tele?neurolog* [TiAb] OR tele?stroke[TiAb] OR Tele?consult*[TiAb] OR Digital?health*[TiAb] OR Tele?visit*[tiab] OR e?Health[Ti] OR e?consult*[Tiab] OR ((Virtual*[Tiab] OR Remote*[tiab]) AND (health[tiab] OR care[tiab])) OR e?visit*[Tiab] OR "electronic visit*"[Tiab] OR outpatient*[Tiab] OR "Video Visit*"[tiab] OR "remote consult*"[tiab]) AND (Outcome Assessment, Health Care[MeSH] OR Quality of Health Care[MeSH] OR Surveys and Questionnaires[MeSH] OR Practice Patterns, Physicians'[MeSH] OR Practice Patterns, Nurses[MeSH] OR Professional patient relations[MeSH] OR Physician Patient Relations[MeSH] OR Nurse Patient Relations[MeSH] OR Job Satisfaction[MeSH] OR Patient satisfaction[MeSH] OR Personal satisfaction[MeSH] OR Patient-Centered Care[MeSH] OR Patient-center*[Tiab] OR User-center*[Tiab] OR (Patients[MeSH] OR Patient*[Tiab] OR Health Personnel[MeSH] OR Provider*[Tiab] OR Physician*[Tiab] OR Nurse*[MeSH] OR Nurse*[Tiab] OR Neurologist*[TiAB] OR "Neurologists"[MeSH] OR Clinician*[tiab] OR User*[tiab] OR Outpatients[MeSH] OR Outpatient*[Tiab] OR Ambulatory) AND (satisfaction*[Tiab] OR experience*[Tiab] OR perspective*[Tiab] OR Evaluation*[Tiab] OR Survey*[Tiab] OR Lesson*[Tiab] OR Practice* [Tiab] OR Observation*[Tiab] OR Attitude*[Tiab] OR View*[Tiab] OR Attitude of Health Personnel[MeSH] OR Attitude to Health[MeSH] ))) AND (Stroke[MeSH] OR Stroke*[Tiab] OR Neurology[MeSH] OR Neurolog*[Tiab] OR "Cerebrovascular Disorders"[MeSH] OR Neurosurgical Procedures[MeSH] OR Neurologic Examination[MeSH] OR Neurologists[MeSH])) NOT (Rehabilitation[MeSH] OR Telerehabilitation[MeSH] OR Rehabilitation[TiAb] OR Telerehabilitation[TiAb] OR Editorial[Publication Type] OR Letter[Publication Type] OR Comment[Publication Type] OR News*[Publication Type] OR Review*[Publication Type] OR Letter[Tiab] OR Comment*[Tiab] OR Systematic Review[Ti] OR Scoping Review[Ti])) AND (journalarticle[Filter] AND 2020/3/1:3000/12/12[pdat] AND English[Filter]))

### CINAHL 174 (April 17, 2021) Filter: Nov 2019 to present (Limiters - Published Date: 20191101-)

(MW ( "Remote Consultation" OR "Telemedicine" OR "Videoconferencing" OR "Telenursing" OR "Telehealth" OR “Ambulatory Care” OR “Outpatients” OR “Distance Counseling” OR “Home Care Services” OR telestroke OR teleneurology ) OR TI ( Tele?medicine OR Tele?health* OR Tele?neurolog* OR tele?stroke OR Tele?consult* OR Digital?health* OR Tele?visit* OR e?Health OR e?consult* OR “Virtual health” OR “Remote care” OR e?visit* OR "electronic visit*" OR outpatient* OR "Video Visit*" OR "remote consult*" ) OR AB ( Tele?medicine OR Tele?health* OR Tele?neurolog* OR tele?stroke OR Tele?consult* OR Digital?health* OR Tele?visit* OR e?Health OR e?consult* OR “Virtual health” OR “Remote care” OR e?visit* OR "electronic visit*" OR outpatient* OR "Video Visit*" OR "remote consult*" )) AND (MW ( "Stroke+" OR "Stroke Units" OR "Stroke Patients" OR neurology OR "Cerebrovascular Disorders" OR “Neurologists” OR “Neurosurgical Procedures” OR “Neurologic Examination” ) OR TI ( "Stroke" OR "Neurolog*" ) OR AB ( "Stroke" OR "Neurolog*" )) AND (MW ( "Quality of Health Care" OR "Patient Preference" OR "Patient Satisfaction+" OR "Personal Satisfaction" OR "Professional-Client Relations+" OR "Professional-Patient Relations" OR "Physician-Patient Relations" OR "Nurse-Patient Relations" OR “Outcome Assessment, Health Care” OR “Surveys and Questionnaires” OR “Practice Patterns, Physicians'“ OR “ Practice Patterns, Nurses“ OR “Job Satisfaction“ OR “Patient-Centered Care” OR “Attitude of Health Personnel” OR “Attitude to Health” ) OR TI ( “Patient-center*” OR “User-center*” OR ((“Patient*” OR “Health Personnel” OR “Provider*” OR “Physician*” OR “Nurse*” OR “Neurologist*” OR “Clinician*” OR “User*”) AND (“satisfaction*” OR “experience*” OR “perspective*” OR “Evaluation*” OR “Survey*” OR “Lesson*” OR “Practice*” OR “Observation*” OR “Attitude*” OR “View*”)) ) OR AB ( “Patient-center*” OR “User-center*” OR ((“Patient*” OR “Health Personnel” OR “Provider*” OR “Physician*” OR “Nurse*” OR “Neurologist*” OR “Clinician*” OR “User*”) AND (“satisfaction*” OR “experience*” OR “perspective*” OR “Evaluation*” OR “Survey*” OR “Lesson*” OR “Practice*” OR “Observation*” OR “Attitude*” OR “View*”)) )) NOT (MW ( “Telerehabilitation” OR “Rehabilitation” ) OR TI ( “Rehabilitat*” OR Telerehabilitat*” OR “Systematic Review” OR “Scoping Review” OR “Rapid Review” OR “Opinion*” ) OR AB ( “Rehabilitat*” OR Telerehabilitat*” OR “Systematic Review” OR “Scoping Review” OR “Rapid Review” OR “Opinion*” ) OR PT ( “Editorial” OR “Letter” OR “Comment*” OR “News*” OR “Review*” OR “Opinion*” ))

### Scopus 381 (April 17, 2021)

( ( INDEXTERMS ( "Stroke" OR "Stroke Units" OR "Stroke Patients" OR neurology OR "Cerebrovascular Disorders" OR "Neurologists" OR "Neurosurgical Procedures" OR "Neurologic Examination" ) OR TITLE-ABS-KEY ( "Stroke" OR "Neurolog*" ) ) AND ( INDEXTERMS ( "Remote Consultation" OR "Telemedicine" OR "Videoconferencing" OR "Telenursing" OR "Telehealth" OR "Ambulatory Care" OR "Outpatients" OR "Distance Counseling" OR "Home Care Services" OR telestroke OR teleneurology ) OR TITLE-ABS-KEY ( telehealth OR telemedicine OR e?health OR e?consultation OR "remote consult*" OR "telephone consult*" OR "" teleconsult* "" OR "" virtual* AND visit* "" OR e?visit* OR "electronic visit*" ) ) AND ( INDEXTERMS ( "Quality of Health Care" OR "Patient Preference" OR "Patient Satisfaction" OR "Personal Satisfaction" OR "Professional-Client Relations" OR "Professional-Patient Relations" OR "Physician-Patient Relations" OR "Nurse-Patient Relations" OR "Outcome Assessment, Health Care" OR "Surveys and Questionnaires" OR "Practice Patterns, Physicians'" OR " Practice Patterns, Nurses" OR "Job Satisfaction" OR "Patient-Centered Care" OR "Attitude of Health Personnel" OR "Attitude to Health" ) OR TITLE-ABS-KEY ( "Patient-center*" OR "User-center*" OR ( "Patient*" OR "Health Personnel" OR "Provider*" OR "Physician*" OR "Nurse*" OR "Neurologist*" OR "Clinician*" OR "User*" ) AND ( "satisfaction*" OR "experience*" OR "perspective*" OR "Evaluation*" OR "Survey*" OR "Lesson*" OR "Practice*" OR "Observation*" OR "Attitude*" OR "View*" ) ) ) ) AND NOT ( INDEXTERMS ( "Telerehabilitation" OR "Rehabilitation" ) OR TITLE-ABS-KEY ( "Rehabilitat*" OR "Telerehabilitat*" OR "Systematic Review" OR "Scoping Review" OR "Rapid Review" OR "Opinion*" ) ) AND ( LIMIT-TO ( SRCTYPE , "j" ) ) AND ( LIMIT-TO ( PUBSTAGE , "final" ) ) AND ( LIMIT-TO ( DOCTYPE , "ar" ) ) AND ( LIMIT-TO ( PUBYEAR , 2021 ) OR LIMIT-TO ( PUBYEAR , 2020 ) ) AND ( LIMIT-TO ( LANGUAGE , "English" ) )

### PsycInfo 34 (April 17, 2021) limit 5 to (peer reviewed journal and english language and yr="2019")

1 (telemedicine or videoconferencing or remote consultation or Ambulatory Care Facilities or Outpatients or Ambulatory Care or Distance Counseling or Home Care Services).mh. or (Tele?medicine or Tele?health* or Tele?neurolog* or tele?stroke or Tele?consult* or Digital?health* or Tele?visit* or e?Health or e?consult* or e?visit* or "electronic visit*" or outpatient* or "Video Visit*" or "remote consult*" or ((Virtual* or Remote*) and (health or care))).ti. or (Tele?medicine or Tele?health* or Tele?neurolog* or tele?stroke or Tele?consult* or Digital?health* or Tele?visit* or e?Health or e?consult* or e?visit* or "electronic visit* " or outpatient* or "Video Visit* " or "remote consult* " or ((Virtual* or Remote*) and (health or care))).ab.

2 (Outcome Assessment Health Care or Quality of Health Care or "Surveys and Questionnaires" or Practice Patterns Physicians or Practice Patterns Nurses or Professional patient relations or Physician Patient Relations or Nurse Patient Relations or Job Satisfaction or Patient satisfaction or Personal satisfaction or Patient-Centered Care or Outpatients or Ambulatory or Attitude of Health Personnel or Attitude to Health).mh. or (Patient-center* or User-center* or ((Patient* or Health Personnel or Provider* or Physician* or Nurse* or Neurologist* or Clinician*OR User*) and (satisfaction* or experience* or perspective* or Evaluation* or Survey* or Lesson* or Practice* or Observation* or Attitude* or View*))).ti. or (Patient-center* or User-center* or ((Patient* or Health Personnel or Provider* or Physician* or Nurse* or Neurologist* or Clinician*OR User*) and (satisfaction* or experience* or perspective* or Evaluation* or Survey* or Lesson* or Practice* or Observation* or Attitude* or View*))).ab.

3 (Stroke or Neurology or Cerebrovascular Disorders or Neurosurgical Procedures or Neurologic Examination or Neurologists).mh. or (Stroke* or Neurolog*).ti. or (Stroke* or Neurolog*).ab.

4 (Rehabilitation or Telerehabilitation).mh. or (Rehabilitat* or Telerehabilitat* or Letter or Comment* or Systematic Review or Scoping Review).ti. or (Rehabilitat* or Telerehabilitat*).ab. or (Editorial or Letter or Comment or News* or Review*).pt.

5 (1 and 2 and 3) not 4

6 limit 5 to (peer reviewed journal and english language and yr="2021")

**Appendix D.** The SERVQUAL questionnaire of a telehealth program in the case hospital conducted by Yin et al. (2016)

| Dimension | Questions |
| --- | --- |
| Tangible | 1. The hospital has easy to operate equipment  2. The hospital provides teaching lessons for equipment operations  3. Physiological measurement equipment provided by the hospital is functioning normally |
| Reliability | 4. Hospital staff has completed the promised duties in time  5. Hospital staff is concerned my problem and resolves my problem  6. Equipment services provided by the hospital are trustworthy and reliable  7. Hospital staff can reply my needs and recommendations in time  8. The hospital can keep my measurement data each time and provide my historical data for query |
| Responsiveness | 9. Hospital staff contacts me directly to make an arrangement for further examination when abnormal physiological measurement data are found  10. My questions have been quickly responded  11. Hospital staff is willing to understand and solve my problems  12. Hospital staff provides the timely services for me |
| Assurance | 13. I feel warm and ease when hospital staff provides services  14. I feel comfortable and trust during the interaction with hospital staff  15. Hospital staff provides services friendly and courteously  16. Services provided by hospital staff are complete and meet my needs |
| Empathy | 17. The hospital can provide customized needs for individuals  18. Hospital staff has sufficient medical knowledge and technology  19. Hospital staff respects opinions and cares needs for individuals  20. The hospital places a high priority for my interests  21. The operation hours provided by the hospital meet my needs |

**Appendix E**. SERVQUAL model codebook.

| Codes | Descriptions | Clinician | Patient |
| --- | --- | --- | --- |
| 1 | **Tangibles** | X | X |
| 1.1 | Comfort level using virtual equipment (subjective) | X | X |
| 1.2 | Technical issues: equipment and capacity /functionality/connectivity/organizational (objective) | X | X |
| 1.3 | Technical support | X | X |
| 1.4 | Environment (setting of the teleconsulting) | X | X |
| 1.5 | Provide training/teaching | X | X |
| 2 | **Reliability** | X | X |
| 2.1 | Administration/scheduling support | X | X |
| 2.2 | Punctual (start on time or duration) | X | X |
| 2.3 | Timing of appointment/referral/DI testing/Rx/lab requisition |  | X |
| 2.4 | Appropriate triage | X |  |
| 2.5 | Chart pre-/documentation readiness | X |  |
| 3 | **Responsiveness** | X | X |
| 3.1 | Address logistical needs (transportation, physical, work, driving, financial etc.) | X | X |
| 3.2 | Response from the clinic (easy to contact) |  | X |
| 3.3 | Address medical needs |  | X |
| 4 | **Assurance** | X | X |
| 4.1 | Virtual verbal communication (sending or receiving information) | X | X |
| 4.2 | Virtual non-verbal verbal communication (emotion, attitude, personality, supplement to verbal communication) | X | X |
| 4.3 | Trust during interaction | X | X |
| 4.4 | Confidence | X | X |
| 4.5 | Clinical component | X |  |
| 4.5.1 | Diagnosis | X | X |
| 4.5.2 | History taking | X |  |
| 4.5.3 | Neurology physical examination/assessment | X |  |
| 4.5.4 | Treatment plan | X |  |
| 4.5.5 | Advanced decision making | X |  |
| 4.5.6 | Image/tests review | X |  |
| 4.5.7 | Documentation | X |  |
| 4.6 | Completeness | X | X |
| 5 | **Empathy** | X | X |
| 5.1 | Personal attention | X | X |
| 5.2 | Human touch | X | X |

**Appendix F**. The most frequent SERVQUAL dimensions, process factors, sub-themes with selected quotes (N=300)

| Dimension | Most frequent process factors | Sub-themes | Selected quotes |
| --- | --- | --- | --- |
| Assurance  (n=113)  (The knowledge and courtesy of employees and their ability to inspire trust and confidence) | Clinical activities (n=69):  PE (n=32) | The positive role of video in PE    Limitation of remote PE;  Lacking utilization of remote assessment tools;  Availability of family support | Better interaction between clinicians and patients, and the use of video to enable a degree of examination led to an improved experience (6_PR^~~b~~^_59).  52% of providers disagreed that they were able to do the relevant neurological examination virtually (14_PR_310).  A significant implementation barrier was the limited nature of the remote neurologic examination (11_PR_371).  A majority of the responders (83.3%) reported not having administered any questionnaires (quality of life, adverse effects, depression, etc.) over the phone or by videoconference. (5_PR_271)  Occasionally required assistance from a caregiver to position the phone to properly observe the patient, perform certain physician-directed exams (16_PR_164) |
|  | Confidence in care  (n=23) | Video adding confidence;  Experiences and training adding confidence;  Perceptions of decreased standard care.  Unusual conditions (delivery bad news or sensitive information) lowing the confidence. | Compared with telephone (audio only), the use of videoconferencing technology was associated with increased confidence in the ability of telemedicine to formulate and communicate a treatment plan when compared to in-person visits (12_PR_296).  Those with neurosurgery training described increased confidence in telemedicine for imaging review and explanation (12_PR_301).  …the corresponding proportion that reported an overall reduced standard of care during the pandemic were, respectively, 9% and 30% in epilepsy, 3% and 42% in headache, 24% and 71% in multiple sclerosis, and 57% and 74% in movement disorders. (10_PR_253). |
|  | Communication  (n=17) | Perceived risk of misunderstanding.  Difficult recognizing emotion;  Difficult establishing trust relationship  Superiority of video visits in communication (enhance PE and diagnosis). | …only 62.5% felt the patient had understood the information correctly. (5_PR_270)  The difficulties recognizing when patients are upset(1_PR_3)  Clinicians reported that video visits were superior to a phone call, allowing them to gather more information than just a medical history. (16_PR_158). |
| Reliability  (n=96)  (the ability to perform the promised service dependably and accurately) | Appropriate triage  (n=55) | Clinical factors:   - Follow-up vs New - Screening or stratification - Disease characteristics   (severity, stability, acuity, complexity)  Patient factors:   - Demographic; - Physical or psychological   limitation; - Caregiver support; - Access to technology; - Experience in using technology. | When asked what type of visit telehealth was most suited for, 62% of providers chose follow-up visits (14_PR_317).  Would want to conduct a traditional appointment if the patient had new or worsening symptoms. (3_PR_91).  Numerous clinicians mentioned that some patients, particularly older adults and lower-income patient populations (e.g., unhoused individuals or rural farm workers), lacked technological capability to support a video call. (16_PR_154)  In the absence of a supportive family member/caregiver, video visits with patients with cognitive, hearing, or visual impairment were also considered nonideal. (16_PR_155) |
|  | Administrative support (n=19) | Change work flow: Scheduling and registration;  Previsit preparation technically and medically;  Accurate patient information. | …administrative challenges in registration and scheduling (11_PR_346).  Patients were often unprepared (6_PR_37)  Lack of previsit charting and medication reconciliation (16_PR_134). |
| Tangible  (n=61)  (The equipment and personnel) | Technical issues  (n=44) | System availability;  System reliability;  System connectivity;  System flexibility;  Functionality limitation. | Barriers noted included access to technology, (16_PR_138)  …challenges in adapting platforms to meet the needs of patients and clinicians (11_PR_345) |
| Responsiveness  (n=23)  (The willingness to help customers and provide prompt service) | Address patients’ logistical needs (n= 13) | Convenience (save time, travel and decrease cost) | Providers felt that televisits would reduce burden on patients related to transportation, behavior, and physical limitations. (3_PR_92)  … increased convenience for patients, less cost for patients…(15_PR_229) |
| Empathy (n=7)  (The provision of individual care and attention to customers) | Human touch  (n=6) | Losing/missing relationship  Lacking empathy: business-like | 38% of clinician Missing/losing (the in-person connection/) relationship with patients (16_PR_115).  …sense of empathy that is challenging to achieve remotely (15_PR_219).  The perception that telephone consultations were of a different style, more ‘business-like’. (6_PR_22) |

**Appendix G.** The most frequent SERVQUAL dimensions, process factors, sub-themes and selected quotes among patients (N=101)

| Dimension | Most frequent process factors | Sub-themes | Selected quotes |
| --- | --- | --- | --- |
| Responsiveness (n=33)  (The willingness to help customers and provide prompt service) | Address logistical needs (n=17) | Convenience (saving time, travel and cost) | Participants considered no transport...no travel expense as advantage of TM appointment. (18_PT_36).  I didn’t have to drive there, find parking, and all the way to the office, wasting an hour plus if time. (9_PT_113).  Eighty-eight percent of patients agreed that their telehealth visit was more convenient for them than an in-person visit.(14_PT_127) |
|  | Address medical needs (n=14) | Address communicative needs (e.g., understanding care plan, or disease, change medication regiments.) | Regarding to the ease of ….understanding the plan-of-care, the most frequent response was “very satisfied" (average rating 5.5/6). (3_PT_33)  (About 88%) Majority of the participants stated that the TM appointment helped them as well as previous appointments onsite for the understanding of the illness, (18_PT_63) |
| Tangible  (n=25)  (The equipment and personnel) | Technical issue  (n=19) | Connectivity;  Usability;  Availability;  Family support. | A lag in audio (or visual) (3_PT_17)  We found that patients experienced MyChart logistical challenges with synchronous TN, which resulted in switching to non-MyChart platforms. (11_PT_178)  ... the lack of PC, tablet, or phone with Internet connection in 8 cases (23.5%) (2_PT_173)  Televisits performed in the presence of subjects of younger generation had a successful rate higher than the group without younger generation caregiver (2_PT_176) |
|  | Home environment (n=6) | Comfort | Participants considered...more comfort as advantage of TM appointment. (18_PT_37)  ‘‘Comfort of being in your own home. (9_PT_101) |
| Assurance  (n=23)  (The knowledge and courtesy of employees and their ability to inspire trust and confidence) | Communication  (n=12) | Situational effectiveness | Language barrier without gesture compensated communication (0.9%) (19_PT_79)  Sixty-four percent of patients agreed that they were able to adequately show their clinical signs to their provider, (14_PT_124) |
|  | Diagnosis (n=5) | Delay and uncertain | Other disadvantages were the postponements of diagnostics or therapies (5.5%) (19_PT_77)  Patients who found virtual clinic to be “not as good” were more likely to have an underlying neurological disorder that would benefit from clinical examination, namely, a neuromuscular condition (66.7%) (2_PT_14) |
| Reliability  (n=15)  (the ability to perform the promised service dependably and accurately) | Tests, prescriptions, Treatments  (n=8) | Delay | Lack of immediate prescription (9%) (18_PT_47).  A delay in performance of epilepsy-related tests occurred in 37 patients (14.5%). Routine EEG was the test most often delayed (n = 11; 29.7%), followed by MRI (n = 9; 24.3%) and video-EEG monitoring (n = 8; 21.6%). (8_PT_133) |
| Empathy  (n=5)  (The provision of individual care and attention to customers) | Personal attention (n=3) | Present  Embarrassing | Felt more present and focused (9_PT_103).  … embarrassing nature of the teleconsultation ... were unfavorable to satisfaction of the patient (4_PT_183). |
