# Supplementary material for: Service process factors affecting patients’ and clinicians’ experiences on rapid teleconsultation implementation in out-patient neurology services during COVID-19 pandemic: a scoping review
Source: BMC Health Serv Res. 2022 Apr 22;22:534. doi: 10.1186/s12913-022-07908-4 (PMC9026006; doi:10.1186/s12913-022-07908-4)
Supplement: Supplementary file 2 — Additional file 2. Raw dataset. [file 12913_2022_7908_MOESM2_ESM.docx]

**Additional file 2.** Raw dataset

| Study # | PT/PR | Content |
| --- | --- | --- |
| 1 | PR | Clinicians most frequently perceived risk in relation the lack of non-verbal communication. |
| 1 | PR | The need to clarify understanding with the paitent (is not met) |
| 1 | PR | The difficulties recognising when patients are upset |
| 1 | PR | Clinicans clearly perceived the ability to observe non-verbal communication as crucial to diagnosing patients accurately |
| 1 | PR | Clinicians were also concerned about the lack of examination. |
| 1 | PR | Without examination findings they felt at risk of missing important signs |
| 1 | PR | Video was not considered a substitiue for F2F examination, at least not in all patients |
| 1 | PR | the lack of non-verbal communication and physical examination resulted in clinicians displaying a lack of confidence during remote consultations |
| 1 | PR | Over one half described a lower level of confidence than in F2F consultations, causing problems with their ability to formulate diagnoses and to discharge patients |
| 1 | PR | Many clinicians felt they were requesting more investigations to clarify questions not answered due to the lack of examination |
| 1 | PR | remote consultations would give patient a better experience by reducing patients’ waiting or travelling especially considering the additional difficulties faced by those with distabilities |
| 1 | PR | Clinicians had perceived their patients as more relaxed during remote consultations than in F2F consultations |
| 1 | PR | Reduce barriers for patients unable to drive themselves to their appointments. Clinicians had perceived their patients as more relaxed during remote consultations than in F2F consultations |
| 1 | PR | Twelve of the clinicians associated an improved efficiency with the length of times patients had to wait, either in the waiting room or for an appointment to see a neurologist |
| 1 | PR | clinicians often commented on how they had adapted their clinic conversation to fit the telephone format and improve the consultations. |
| 1 | PR | the perception that telephone consultations were of a different style, more ‘business-like’. |
| 1 | PR | Clinicians commented on how they were able to “take control” of and to guide the consultations. |
| 1 | PR | This added control was presented as an advantage over traditional F2F clinic encounters. |
| 1 | PR | Technical issues were the most pominent challenge, mentioned in 20/22 interviews. |
| 1 | PR | Technical issues were partly due to the limitations of the technology available and partly to how tele-neurology was implemented. |
| 1 | PR | Due to policies intended to reduce hospital footfall and reduce potential COVID-19 exposure of administrative staff during the pandemic, clinicians were without paper patient notes during the initial implementation phase of tele-neurology. |
| 1 | PR | Clinicans had to rely on existing software to retrieve old letters or look up investigation findings. |
| 1 | PR | For most clinicians this change highlighted the inadequency of their information technology systems |
| 1 | PR | Clinicians explained how the introduction of tele-neurology, particularly video consultations, demonstrated their hospital was unable to provide adequate technology support to use the available equipment and software to its full potential |
| 1 | PR | More technical issues were reported utilising video conferencing software than telephone technology |
| 1 | PR | The administration of tele-neurology clincs provided further frustration for 18 of the clinicians. |
| 1 | PR | Clinicians described how it was not uncommon for the patient contact number to be missing, or for an incorrect number to have been provided |
| 1 | PR | This resulted in clinicians spending their clinic time searching for patient contact details across multiple platforms. |
| 1 | PR | Clinicians believed patients were often unprepared for a telephone or video consultations, attributable to the failure of the clinic booking team to get in touch with patients prior to their appointments. |
| 1 | PR | clinicians highlighted the importance of not only contacting patients prior to clinic, but of preparing them better for their consultations |
| 1 | PR | Clinicians perceived some specific groups of patients as particularly challenging to assess in remote consultations |
| 1 | PR | Concerns were also voiced about consultations in which difficult decisions had to be made because clinicians perceived they had to be more direct to advance decision-making over the telephone and they were less able to take account of patients’ own views. |
| 1 | PR | a greater risk of misunderstandings over the telephone with select patient groups. |
| 1 | PR | Furthermore, new patients were frequently perceived as difficult to manage remotely. |
| 1 | PR | (for FU) the greater ease with which rapport may be built in F2F consultations. |
| 1 | PR | This limited ability to build rapport was also described as posing a challenge in encounters involving the delivery of bad news to patients. |
| 1 | PR | Twelve clinicians described how breaking bad news to patients via tele-neurolgy made them feel uncomfortable |
| 1 | PR | Being unable to peform these tests, and progress in the management of conditions, could cause clinicians to feel the consultation was unfinished. |
| 1 | PR | The main reason for this seemed to be their concerns about the lack of interaction with patients, although 6/22 clinicians noted this was mitigated to some extent by the use of video-clinics. |
| 1 | PR | Despite the challenges clinicians have experienced adopting tele-neurology, all 22 clinicians anticipate tele-neurology will continue to be utilised. |
| 1 | PR | Whilst highlighting that video is not a substitue for F2F consultations |
| 1 | PR | 16 of the clinicians described situations in which video benefited their remote consultations. |
| 1 | PR | When not disrupted by technical issues, video was viewed as superior to telephone on many occasions. |
| 1 | PR | Better interaction between clinicians and patients, and the use of video to enable a degree of examination led to an improved experience |
| 1 | PR | Importantly, six of the clinicians commented on how utilising video had improved their job satisfaction |
| 1 | PR | When asked about the utilisation of tele-neurology in the future, clinicians felt tele-neurology has obvious benefits in follow- up appointments, particularly of patients with a well-established diagnosis requiring regular monitoring. |
| 1 | PR | Remote consultations with patients with epilepsy were highlighted as a particular succes of tele-neurology, at least from the clinicians’ perspective |
| 1 | PR | In contrast to follow-up patients, clinicians felt remote consultations were inappropriate for new patients |
| 1 | PR | Half of the clinicians thought tele-neurology will have an important role in patient triage (or stratification). |
| 1 | PR | Half of the clinicians thought tele-neurology will have an important role in patient (triage or) stratification |
| 1 | PR | The suggestion was also made that patients could initally be contacted remotely via tele-neurology, and that only pre-selected patients would be seen F2F. |
| 1 | PR | This suggestion (initial patient teleneurology screen for F/F) reflected clinicians concerns about service pressures and increasingly long waiting times within their department. |
| 1 | PR | Clinicians perceived changes to the delivery of healthcare are necessary in the longer term, by reflecting on how their existing practice was not environmentally sustainable. |
| 1 | PR | Focus group clinicians strongly emphasised the administrative problems they face in their routine practice and how these have interacted negatively with the enforced introduction of tele-neurology |
| 1 | PR | These comments reflected perceptions of insufficient staffing as well as (infrastructural deficits) undermining an optimal adoption of tele-neurology. |
| 1 | PR | These comments reflected perceptions of (insufficient staffing as well as)infrastructural deficits undermining an optimal adoption of tele-neurology. |
| 1 | PR | how better clinic planning was particularly important for patients with hearing impairments, for whom tele-neurolgy appointments may not be appropriate |
| 1 | PR | some clinicians re-stating video-encounters being more enjoyable than telephone conversations. |
| 1 | PR | when not affected by technical issues, was regarded a success with clinicians feeling better able to formulate new diagnoses using this modality. |
| 1 | PR | clinicians reiterated that breaking bad news to patients via tele-neurology was not acceptable and |
| 1 | PR | (clinicians reiterated that breaking bad news to patients via tele-neurology was not acceptable and) emphasised the importance of unimpeded nonverbal communication. |
| 1 | PR | Patients with functional neurological disorder or dissociative seizures were named as being especially difficult to advise successfully using tele-neurology |
| 1 | PR | Concerning the future, clinicans commented on how tele- neurology may be a useful screening tool allowing services to manage some patients remotely |
| 1 | PR | it was felt there was a definite place for tele-neurology in the management of patients with relatively stable and predictable long-term or chronic neurolgoical conditions. |
| 1 | PR | A new idea outlined the potential of tele-neurology to support new ways of collabora- tion between general practitionners, neurologists and patients or their relatives |
| 2 | PT | the majority of patients stated that their neurological condition was stable during the COVID-19 pandemic (84.5%) |
| 2 | PT | a small number stated that it was worse (6.8%) |
| 2 | PT | a small number thought that it was better (6.8%) |
| 2 | PT | Those who deemed that their status was better were more likely to have epilepsy than any other diagnosis |
| 2 | PT | Regarding ease of contacting the neurology service, almost all patients found it was either easy to contact (56.9%) or that they did not need to make contact (41.7%) |
| 2 | PT | a few patients found it difficult to make contact (1.4%) |
| 2 | PT | For the most part, patients had not been advised to attend the hospital (88.1%) |
| 2 | PT | a substantial proportion of those who had been advised to attend the hospital were too afraid to do so (40%) |
| 2 | PT | About three-quarters of our patients found remote consultations either “just as good” (67.1%) than face to face conslation. |
| 2 | PT | small numbers of patient found remote consulattions "better" (9%) than face to face conslatation. |
| 2 | PT | Those who deemed “not as good” were significantly older (approximaly 6 years older) |
| 2 | PT | For (those who deemed "not as good"), there was no difference in sex. |
| 2 | PT | Patients who found virtual clinic to be “not as good” were more likely to have an underlying neurological disorder that would benefit from clinical examination, namely, a neuromuscular condition (66.7%) |
| 2 | PT | (Patients who found virtual clinic to be “not as good” were more likely to have) an undiagnosed condition awaiting investigation or clinical review. |
| 3 | PT | difficulties hearing the provider |
| 3 | PT | a lag in audio (or visual) |
| 3 | PT | a lag in (audio or) visual |
| 3 | PT | not receiving the email link. |
| 3 | PT | the majority did not seem to have issues (technical difficulties) |
| 3 | PT | 17.6% did report difficulties (technical) |
| 3 | PT | A minority of participants, 20.5%, reported transportation issues with traditional visits. The reasons include long commute, reliance on family members, or need to use commercial or alternative (e.g., Uber) transportation. |
| 3 | PT | Participants who reported behavioral issues including autism or postictal confusion were 5.9% |
| 3 | PT | Physical limitations affected 4.4% of participants' ability to come to a traditional visit. The physical limitations that impacted coming to clinic include need for an assistive device. |
| 3 | PT | The physical limitations that impacted coming to clinic included seizure frequency. |
| 3 | PT | Participants reported financial troubles related to attending traditional visits such as taking time off work, childcare needs, and job loss related to time off. |
| 3 | PT | Financial issues affect 22.1% of the participants. |
| 3 | PT | Only 4.4% of patients reported difficulties utilizing the technology necessary for telehealth visits including poor cell phone connection and lack of video app. |
| 3 | PT | All the participants were comfortable with the technology needed to use the video apps. |
| 3 | PT | Regarding ease of scheduling, checking in, …..and scheduling a follow-up, the most frequent response was “very satisfied" (average rating 5.5/6). |
| 3 | PT | Regarding to stability of connection, (the most frequent response) was “very satisfied" (average rating 5.5/6). |
| 3 | PT | Regarting to the ease of ….understanding the plan-of-care, the most frequent response was “very satisfied" (average rating 5.5/6). |
| 3 | PT | The survey asked how likely the patient would be to choose a televisit over a traditional visit. ….66% participants answered 6 (very likely). |
| 3 | PR | Two providers saw delay in reaching patients, either related to a technical issue (or related to the patient not being available at the time of appointment). |
| 3 | PR | Two providers saw delay in reaching patients, (either related to a technical issue or) related to the patient not being available at the time of appointment. |
| 3 | PR | Only one provider used interpretation services via telephone in conjunction with the telehealth visit and reported no difference from a traditional clinic visit. |
| 3 | PR | Regarding connectivity issues, the most frequent answer was satisfied (5 in a range of 1–6). |
| 3 | PR | All providers answered a 6 for being able to maintain patient privacy during the visits. |
| 3 | PR | All providers felt comfortable using the technology. |
| 3 | PR | Most of the providers found it useful in increasing compliance with visits (and felt that this was something they could use in the future). |
| 3 | PR | Most of the providers (found it useful in increasing compliance with visits and) felt that this was something they could use in the future. |
| 3 | PR | providers felt that televisits would reduce burden on patients related to transportation, behavior, and physical limitations. |
| 3 | PR | Majority (67%) of provider indicated that the choosing telehealth visit depends on scenario. (figure 6: 33% chosen telehealth visit only) |
| 3 | PR | 33% of provoder will choose telehealth visit in the furture. (figure 6) |
| 4 | PT | (82%) Majority participants were satisfied with their TM appointment. |
| 4 | PT | Participants considered no transport..no travel expance as advantage of TM appointment. |
| 4 | PT | Participants considered ..more comfort as advantage of TM appointment. |
| 4 | PT | Participants considered ...short waiting time... as advantage of TM appointment. |
| 4 | PT | Ability in better following the conversation (6%) (and preparing for the appointment (12%) were) was considered less important |
| 4 | PT | Ability in ... preparing for the appointment (12%) were considered less important |
| 4 | PT | participants (8%) stated spontaneously that TM appointments avoid or reduce the infection risk. |
| 4 | PT | Participants considered the lack of personal contact (44%) [and of further diagnostics (45%)] as disadvantages of TM appointments. |
| 4 | PT | Participants considered [the lack of personal contact (44%) and] of further diagnostics (45%) as disadvantages of TM appointments. |
| 4 | PT | Leaving habitual surrounding (7%),…. were less important to them. |
| 4 | PT | meeting other patients with epilepsy (9%),…. were less important to them. |
| 4 | PT | .. lack of immediate prescription (9%),.. Was less important to them. |
| 4 | PT | ...as well as technical (10%)..was less important to them |
| 4 | PT | ….or cognitive problems (3%)…was less important to them |
| 4 | PT | The majority of participants would be willing to attend future TM appointment. |
| 4 | PT | However, they wished further appointments onsite (178 patients, 74%) as well (47% at least once a year, 26% on demand). |
| 4 | PT | younger age, not being native German speaker, and shorter duration of being patient at the department were positively associated with the willingness to attend future TM appointments |
| 4 | PT | whereas longer duration of epilepsy was found to be a negative predictor for the wish of future TM appointments |
| 4 | PT | positive predictors for the wish for future appointments onsite were longer duration of epilepsy, taking antiseizure drugs, as well as longer duration of being a patient at our department. |
| 4 | PT | Eighty percent of all participantswished low frequent or on demand onsite appointments in the future, which was significantly associated with the willingness to have TM appointments. |
| 4 | PT | Satisfaction with TM appointment was rated as good as with appointments onsite by 80% of the patients. |
| 4 | PT | equivalence was attested concerning time given by the physician(91%) |
| 4 | PT | equivalence was attested concerning comprehensibility of counseling (91%) |
| 4 | PT | equivalence was attested concerning opportunity to get answers to current questions (92%). |
| 4 | PT | (About 88%) Majority of the participants stated that the TM appointment helped them as well as previous appointments onsite for the understanding of the illness, |
| 4 | PT | Majority (82%) stated that they would follow the advice in the same way as if they had attended the appointment onsite |
| 5 | PR | Prior COVID, (63.4%) had already experienced using a personal (84/109, 77.1%) or an institutional (89/109, 81.7%) remote system. 58.7% fpr patient direct care. |
| 5 | PR | (44.2%), this experience was at least monthly using institutional remote system prior COVID. |
| 5 | PR | The duration of remote clinics was considered as identical as face-to-face in 31.1%, shorter in 47.8% and longer for 21.1% of responders. |
| 5 | PR | Regarding antiseizure medication changes, 50.9% of respondents tended to make fewer amendments, 45.4% the same, 3.7% more. |
| 5 | PR | Electroencephalogram (EEG) were less frequently requested for 65.6%., |
| 5 | PR | Electroencephalogram (EEG) were less frequently requested without change for 30.1%. |
| 5 | PR | Electroencephalogram (EEG) were less frequently requested was more for 4.3%. |
| 5 | PR | Blood test were less frequently requested for 52.8% |
| 5 | PR | blood tests without changing the frequency requested for 42.3%, |
| 5 | PR | blood test were more frequently requested for 4.9% |
| 5 | PR | Respondents reported an increase in email and phone contacts by patients and their families (67.4% and 104, 60.5% of respondents, respectively) but also by primary care physicians (26.2% & 37.2%) |
| 5 | PR | Sixty-one percent of respondents were satisfied by their remote clinics. |
| 5 | PR | Feelings regarding family and patient satisfaction with the remote clinic were positive for 72.2% of the respondents. |
| 5 | PR | 14.3% responders reported dissatisfaction with remote clinics. |
| 5 | PR | Respondents indicated they would likely continue greater use of remotework for remote clinics …..after the COVID- 19 pandemic, in 81.2%. |
| 5 | PR | remote clinics had the advantage of decreasing time and cost for families and patients travel and consequently of work absenteeism. |
| 5 | PR | This was highlighted for follow-up visits but not for new patients having their first evaluation. |
| 5 | PR | For first visits, respondents declared a clear need for a face-to-face visit. |
| 5 | PR | the number of respondents with an institutional remote work system was higher in China (65.5%) |
| 5 | PR | The use of personal remote systems was also significantly different between these countries (p = 0.0002), more frequent in China. |
| 5 | PR | only the impression on families' and patients' satisfaction for remote clinics had a tendency to be higher in France compared with China. |
| 6 | PT | One patient (0.4%) insisted on an urgent appointment because of an increased seizure frequency and seizure clusters, but failed to appear on the arranged day. The patient was seen later at the emergency department. |
| 6 | PT | (this patient who require urgent appointment went to ER had) electroencephalography (EEG) was performed |
| 6 | PT | ( this patient who require urgent appointment went to ER) and (had) antiseizure drugs (ASDs) changed |
| 6 | PT | The subjective personal threat from SARS-CoV-2 for patients and their families was categorized as very serious in 5.5% of subjects, serious in 25.7%, less serious 37.5% , nonexistent in 14.3%. |
| 6 | PT | The most frequent reasons for seeking medical advice were general disease-specific questions and aspects |
| 6 | PT | followed by concerns about side effects of ASDs (31.2%) |
| 6 | PT | ongoing or planned changes in ASD regimens (29.4%) |
| 6 | PT | ASD management during a planned, new or known pregnancy (each 9.2%, |
| 6 | PT | questions about epilepsy-specific driving restrictions (6.4%) |
| 6 | PR | According to physicians' notes, the following issues were addressed in detail during telemedicine consultations: changes or maintenance of ASD regimens (82.6%) |
| 6 | PR | According to physicians' notes, the following issues were addressed in detail during telemedicine consultations: general disease-specific questions and aspects (72.5%) |
| 6 | PR | According to physicians' notes, the following issues were addressed in detail during telemedicine: consultationsSARS-CoV-2-associated questions (40.4) |
| 6 | PR | According to physicians' notes, the following issues were addressed in detail during telemedicine: seizure frequency (35.8%) |
| 6 | PR | According to physicians' notes, the following issues were addressed in detail during telemedicine: side effects of ASDs (34.9%) |
| 6 | PR | According to physicians' notes, the following issues were addressed in detail during telemedicine: ASD prescriptions (33.0%) |
| 6 | PR | According to physicians' notes, the following issues were addressed in detail during telemedicine: social aspects and supportive services or further diagnostic/therapeutic steps (30.3%) |
| 6 | PR | According to physicians' notes, the following issues were addressed in detail during telemedicine: the need for a written change in medication regime (25.7%) |
| 6 | PR | According to physicians' notes, the following issues were addressed in detail during telemedicine: work or employment issues (11.0%) |
| 6 | PR | According to physicians' notes, the following issues were addressed in detail during telemedicine: epilepsy-specific driving restrictions (9.2%) |
| 6 | PT | Most participating patients rated telemedicine appointments as either completely satisfying (38.5%) or satisfying (56.9%) |
| 6 | PT | The conversion from face-to-face appointments to telemedicine appointments was rated as not being a disadvantage for current treatment in 87.2%. |
| 6 | PT | Other disadvantages were the postponements of diagnostics or therapies (5.5%) |
| 6 | PT | limited possibilities for interpretation of ASD side effects or other symptoms (5.5%) |
| 6 | PT | language barrier without gesture compensated communication (0.9%) |
| 6 | PT | increased uncertainty due to lack of face-to-face contact (0.9%) |
| 6 | PR | From the perspective of counseling physicians, 46.8% of appointments were completely satisfying, 45.0% were satisfing |
| 6 | PR | From the perspective of consuling physicians, no telemedicine appointmentwas rated as unsatisfying. |
| 6 | PR | In cases of discrepancies, physicians mostly rated the urgency for the appointment lower than the patients themselve. |
| 6 | PT | Univariate analyses of factors associated with the request of telemedicine consultation rather than a postponed face-to-face visit showed significant associations to: subjectively high-hazard estimations of potential SARS-CoV-2 threats |
| 6 | PT | Univariate analyses of factors associated with the request of telemedicine consultation rather than a postponed face-to-face visit showed significant associations to: subjectively urgent or very urgent medical needs |
| 7 | PT | Migraine patients were significantly more likely to report running out of medications than those with other diagnoses |
| 7 | PT | (Migraine patient) More avoided seeking medical help for new health problems because of the pandemic |
| 7 | PT | A majority of these patients found telemedicine to be easy to use |
| 7 | PT | A majority of these patients found telemedicine ... as valuable as an in-person visit. |
| 7 | PT | a majority would prefer or consider telemedicine for future appointments over in-person visits. |
| 7 | PT | An increase in headache severity or frequency was reported in 39 (33%) out of 118 survey respondents, |
| 7 | PT | Migraine p atients were also significantly impacted economically by the pandemic: 32.4% of migraine patients reported losing their jobs as the result of the pandemic versus (14.5%) of their peers. |
| 7 | PT | respondents with migraine were significantly more likely than their non-migraine peers to report worsening anxiety and sleep problems |
| 8 | PR | initial video visit to require a full examination. |
| 8 | PR | During the 8-week implementation period, video visit adoption was high based on both percentage of clinicians using them, and percentage of visits completed via video |
| 8 | PR | Wihtin the first two weeks, (79%) clinicians integrated video into their practice, increasing to 65 (98%) clinicians by week 6. |
| 8 | PR | Almost all (92%) visits were conducted via video and adoption of video visits was high for both new and return patient visits (93% ). |
| 8 | PR | 63% of clincian reported technological limitations |
| 8 | PR | 38% of clinician Missing/losing the in-person connection/relationship with patients |
| 8 | PR | Difficulties arranging (and completing) necessary follow-ups after the video visit |
| 8 | PR | Difficulties (arranging and) completing necessary follow-ups after the video visit |
| 8 | PR | 15% Patients’ unwillingness to come into clinic for requested in-person visits in the future |
| 8 | PR | 6% patient expectations to have video visits as an option |
| 8 | PR | 4% maintaining access to readily available technology and equipment needed for video visits. |
| 8 | PR | Several video functionalities were suggested for a successful visit, including the following: (1) screen sharing to facilitate patient education and explain imaging results, |
| 8 | PR | Several video functionalities were suggested for a successful visit, including the following (2) a waiting room function to replicate “stepping out of the room” when engaging with trainees, |
| 8 | PR | Several video functionalities were suggested for a successful visit, including the following (3) a chat box for troubleshooting, |
| 8 | PR | Several video functionalities were suggested for a successful visit, including the following (4) file sharing capabilities, |
| 8 | PR | Several video functionalities were suggested for a successful visit, including the following (5) screenshot capabilities to support efficient charting, |
| 8 | PR | Several video functionalities were suggested for a successful visit, including the following (6) multiperson teleconferencing to include other members of the multidisciplinary team, interpreters, trainees, and family members in different physical locations. |
| 8 | PR | Major issues included rigid video visit scheduling, |
| 8 | PR | Major issues included note-taking efficiency. Note-taking was considered easier by some during video visits, reducing after-hours charting time. |
| 8 | PR | (Major issue included) previsit planning. |
| 8 | PR | Several clinicians mentioned that, provided there were no technological issues |
| 8 | PR | (Several clinicain mentioned that) , video visits helped them stay on schedule. |
| 8 | PR | However, hard time limits set by the video visit platform caused frustration. Clinicians could not notify the next patient when running late nor initiate the next visit earlier than scheduled as they might do at the clinic. |
| 8 | PR | Others were unable to document simultaneously, making them less efficient. Dictation was considered a possible solution. |
| 8 | PR | most surveyed clinicians (n=34; 71%) agreed or strongly agreed that video visits allowed them to shift uncompensated to compensated work (ie, scheduling a video visit to address concerns that would previously have been managed through either EMR messaging or unscheduled and uncompensated phone calls). |
| 8 | PR | The lack of integration of medical assistants (MAs) in the video visit workflow, resulting in a lack of previsit charting and medication reconciliation, was another concern. |
| 8 | PR | even involving the patient in their own previsit preparation, was deemed necessary for video visits to be sustained. |
| 8 | PR | Clinicians highlighted several benefits of video visits for patients and families/caregivers, including convenience, impact on travel and cost, (and seeing the patient’s home environment.) |
| 8 | PR | Clinicians highlighted several benefits of video visits for patients and families/caregivers, including (convenience, impact on travel and cost, and) seeing the patient’s home environment. |
| 8 | PR | Barriers noted included access to technology, |
| 8 | PR | patient’s technological capacity, |
| 8 | PR | and language. |
| 8 | PR | Video visits were considered advantageous and convenient, saving patients time and money, particularly for older adults and those who travel long distances for appointments. |
| 8 | PR | Survey respondents (77%) agreed that saving patients unnecessary travel was one of the top 3 benefits. |
| 8 | PR | Several clinicians supported referring patients to local laboratories to further avoid unnecessary travel. |
| 8 | PR | Clinicians also noted that for patients who required assistance (eg, patients with dementia, epilepsy, or mobility issues), the video visit alleviated the travel burden on families/caregivers. |
| 8 | PR | Several clinicians saw value in seeing patients in their home. |
| 8 | PR | (seeing patient in their own home) allow more family involvement. |
| 8 | PR | clinicians could also troubleshoot daily functioning issues or modify the patient’s environment by directly assessing fall hazards, medications, and home devices |
| 8 | PR | increased access to vulnerable populations was rated as one of the top 3 benefits of video visits |
| 8 | PR | numerous clinicians mentioned that some patients, particularly older adults and lower-income patient populations (eg, unhoused individuals or rural farm workers), lacked the necessary access to technology |
| 8 | PR | numerous clinicians mentioned that some patients, particularly older adults and lower-income patient populations (eg, unhoused individuals or rural farm workers),( lacked the necessary access to technology and) technological capability to support a video call. |
| 8 | PR | In the absence of a supportive family member/caregiver, video visits with patients with cognitive, hearing, or visual impairment were also considered nonideal. |
| 8 | PR | Language barriers were also considered a possible limitation of video visit utilization by both interviewees and survey respondents |
| 8 | PR | (35%) surveyed clinicians indicated inclusion of interpreters was one of their top 3 concerns |
| 8 | PR | Clinicians reported that video visits were superior to a phone call, allowing them to gather more information than just a medical history. |
| 8 | PR | Although exam needs varied by clinician and subspecialty, several clinicians described that despite challenges they were pleasantly surprised to be able to perform several modified physical examinations over video. |
| 8 | PR | many stated that the inability to perform a hands-on physical examination was a limitation of video visits that was best paired with a timely in-person follow-up. |
| 8 | PR | Several clinicians mentioned that virtual exams were more time-consuming |
| 8 | PR | occasionally required assistance from a caregiver to position the phone to properly observe the patient, perform certain physician-directed exams, confirm what the patient says, or catch the patient if s/he is at risk of falling. |
| 8 | PR | Additional limitations were related to the patient’s immediate environment, including adequate space for the patient to move around, and sufficient lighting. |
| 8 | PR | Camera positioning was also critical. |
| 8 | PR | Clinicians also noted that occasionally patients took their video call at inappropriate times (eg, while working or driving), despite previsit counseling. |
| 8 | PR | Clinicians considered video visits beneficial to ensure continuity of care for chronic conditions. |
| 8 | PR | There was general agreement that video visits are best suited for established patients, especially those who are stable/uncomplicated, or for a quick checkup without extensive examination or testing. |
| 8 | PR | Although several clinicians felt that most patients were appropriate for video visits, the majority agreed that new patient visits, and patients with acute conditions and declining health, were less suited due to relative ease of the complete physical examination. |
| 8 | PR | Many preferred to first see the patient in person before determining whether further follow-up care can be provided, at least partly, over video. |
| 8 | PR | Clinicians also recognized that some patient populations encounter significant barriers to attending the clinic in person and that video offers an opportunity to continue care. |
| 8 | PR | Most clinicians were positive toward video visits and believed they could incorporate video into their practice long-term, |
| 8 | PR | although several insisted that video cannot replace a full in-person examination. |
| 8 | PR | 40 (83%) surveyed clinicians agreed or strongly agreed that video visits supported their overall well-being. |
| 8 | PR | patient video visits will need to be selected carefully to optimize care and respect preferences |
| 8 | PR | Patients’ suitability for video visits would need to be determined during scheduling based on several criteria (eg, physical examination needs, patient’s technological capacity and demographics, new versus return). |
| 8 | PR | Seldom was the video visit itself considered a good tool for triage. |
| 8 | PR | Most survey respondents (n=39; 81%) agreed that video visits should be supplemented with in-person visits |
| 8 | PR | The recommended frequency of supplemental in-person visits varied among the respondents: 6 (15%) recommended quarterly, 11 (28%) biannually, 17 (44%) annually, and 5 (13%) every 2 years. |
| 8 | PR | concern mentioned by a minority of interviewees and 7 (15%) surveyed clinicians was that patients may find video too convenient and opt out of recommended in-person visits |
| 8 | PR | (19%) survey respondents with representation from epilepsy, memory, headache, stroke, and neuro-oncology, indicated that an all-video practice would be feasible for their patient population. |
| 9 | PT | More than 97% of patients were very highly (83%) or highly (14.3%) confident in the telehealth care they received. |
| 9 | PT | 89.2% of patients rated the telehealth visit as excellent (68.7%) or very good (20.5%) for meeting their medical needs. |
| 9 | PT | Compared with new patients, returning patients had 1.9 times greater odds to report greater satisfaction with the telehealth visit meeting their medical needs |
| 9 | PT | Compared with new patients, returning patients had 1.9 times greater odds to report greater satisfaction with the telehealth visit meeting their medical needs and 1.5 times greater odds to recommend telehealth to their friends or family. |
| 9 | PT | Furthermore, patients avoided an average of 185.08 km, or 2.08 h, of round-trip travel by utilizing teleneurology services. |
| 9 | PT | ‘‘Personally, it’s easier and more comfortable being home (not fighting traffic or looking for parking. Definitely would love to keep this up).’’ |
| 9 | PT | ... not fighting traffic or looking for parking. Definitely would love to keep this up.’’ |
| 9 | PT | ‘‘Comfort of being in your own home. |
| 9 | PT | Felt supported and secure with the interaction with the providers. |
| 9 | PT | Felt more present and focused. |
| 9 | PT | ‘‘Telehealth is such a blessing to people with a chronic disease that causes fatigue! |
| 9 | PT | "….I had hoped for a long time but this practice could offer it. I will use it happily in the future if offered" |
| 9 | PT | ‘‘I have an established history with the physician (and I appreciate being able to stay safe at home and still get the care needed.)" |
| 9 | PT | ‘‘(I have an established history with the physician and) I appreciate being able to stay safe at home and still get the care needed." |
| 9 | PT | ‘‘(I have an established history with the physician and) I (appreciate being able to stay safe at home and) still get the care needed." |
| 9 | PT | ‘‘I loved it." |
| 9 | PT | I would like to have all my follow up visits this way. |
| 9 | PT | The actual time with the doctor was only 10–15 min which is appropriate. |
| 9 | PT | I didn’t have to drive there, find parking, and all the way to the office, wasting an hour plus if time. |
| 9 | PT | This option would literally allow me to accept just about any appointment time as I can do it right from my office and get back to my day. |
| 9 | PT | ‘‘I am so glad you have this system, and hope that we can use it when appropriate even after the distancing discipline has ended. It is a most efficient use of everyone’s time!’’ |
| 9 | PR | ‘‘Despite some occasional connection issues that were mostly easily resolved" |
| 9 | PR | "I think telehealth is a critical resource for our patients that we need to offer going forward.’’ |
| 9 | PR | ‘‘Telehealth must become part of our regular form of care going forward.’’ |
| 9 | PR | ‘‘Telehealth was of greatest utility for established patients and routine followup. For newer patients or returns with reported physical changes, the exam is more limited, which is why I answered the question about recommending telehealth as ‘somewhat likely.’’ |
| 9 | PR | For newer patients or returns with reported physical changes, the exam is more limited, |
| 9 | PR | ‘‘I feel that telehealth was extremely valuable during this difficult time. Patients were very appreciative. |
| 9 | PR | ‘‘I feel that providing Telehealth options in the future would be beneficial, especially for patients who travel’’ |
| 9 | PR | When asked about their audiovisual connections, 88.5% of clinicians could always (13.5%) or most of the time (75%) see the patient without any difficulty, |
| 9 | PR | whereas 92.7% could always (19.8%) or most of the time (72.9%) hear the patient without any difficulty. |
| 9 | PR | In addition, 82.3% of clinicians had very high (37.5%) or high (44.8%) confidence in the care they provided through telehealth, |
| 9 | PR | whereas 98.9%of clinicians were very likely (70.8%) or somewhat likely (28.1%) to recommend telehealth to other clinicians. |
| 10 | PR | The teleconference/video conference visits were used most commonly for postoperative follow-up. |
| 10 | PR | New consultation and surgical discussion were also frequent reasons for teleconference or video conference visits |
| 10 | PR | The duration of teleconference or video conference visits ranged from <15minutes to approximately 30-60 minutes with 78% of participants reporting 15-30 minutes per visit. |
| 10 | PR | Compared with in-person visits, 71% thought that teleconference or video conference visits were shorter; only 1 respondent thought it took longer |
| 10 | PR | Of the 14 participants, 10 thought that teleconference/video conference visits were inferior to traditional in-person visits |
| 10 | PR | Even with the numerous limitations and barriers, 12 of 14 respondents planned to continue using teleconference/video conference visits after COVID-19 restrictions are lifted |
| 10 | PR | Uniformly, all surgeons and APPs noted the inability to perform a neurological examination as a limitation compared with in-person visits. |
| 10 | PR | Furthermore, multiple neurosurgeons raised the concern of being able to establish a meaningful rapport with patients through telemedicine. |
| 10 | PR | In particular, discussing a potentially life-altering diagnosis and treatment plan with major risks (requires a level of trust and sense of empathy that is challenging to achieve remotely). |
| 10 | PR | In particular, (discussing a potentially life-altering diagnosis and treatment plan with major risks) requires a level of trust and sense of empathy that is challenging to achieve remotely. |
| 10 | PR | In particular, (discussing a potentially life-altering diagnosis and treatment plan with major risks requires a level of trust and) sense of empathy that is challenging to achieve remotely. |
| 10 | PR | In such cases, surgeons felt the need to schedule an in-person clinic visit following the telemedicine encounter. |
| 10 | PR | Concerning the technical barriers, surgeons reported challenges in accessing patient imaging during the virtual encounters if the scans were performed inother institutions or the patients failed to mail the hard copy in time. |
| 10 | PR | Even with accessible imaging, some surgeons found reviewing them with the patients over video conference was challenging, (especially ensuring that patients understood both the images and the didactic information). |
| 10 | PR | Even with accessible imaging, (some surgeons found reviewing them with the patients over video conference was challenging,) especially ensuring that patients understood both the images and the didactic information. |
| 10 | PR | This challenge can be further amplified when dealing with inconsistent video and audio connections (along with patients who are unfamiliar with digital communication technology). |
| 10 | PR | This challenge can be (further amplified when dealing with inconsistent video and audio connections along with) patients who are unfamiliar with digital communication technology. |
| 10 | PR | Other common barriers included ... lack of technical support |
| 10 | PR | The perceived strengths of teleconference/video conference visits included increased convenience for patients, less cost for patients, (improved access for surgeons to a broadercatchment area, and cost savings for hospitals). |
| 10 | PR | the teleconference/video conference visits, 93% of respondents relied on clinical support staff as their technical support compared with 64% using the information technology help desk. One respondent reported not having sufficient technical support. |
| 11 | PR | Telephone and video consultations were deemed equally satisfactory from a professional perspective. |
| 11 | PR | Typically, less time was spent using telemedicine compared to in-person appointments. |
| 11 | PR | There was no significant difference between residents and senior consultants regarding the proportion that experienced telephone and video consultations effective and better than regular in-person appointments (28% vs. 39%, P = 0.22). |
| 11 | PR | No significant difference was found between residents and senior consultants regarding the statement “from a professional perspective, follow-ups by telephone consultation do work satisfactorily” (87% vs. 84%, P = 0.62). |
| 11 | PR | More women than men experienced telephone and video consultations effective and better than regular inperson appointments (44% vs. 22%, P = 0.02).This finding was consistent in the multivariable analysis adjusting for age and hospital type (odds ratio 2.61, 95% CI 1.05; 6.49, P = 0.039) |
| 11 | PR | There was a significant difference between women (91%) and men (78%, P = 0.046) regarding the statement “from a professional perspective, follow-ups by telephone consultation do work satisfactorily”. However, this difference was no longer statistically significant after adjusting for age and hospital type |
| 11 | PR | Sixty-one percent of the neurologists within headache care reported that telephone consultations with new referrals worked satisfactorily, compared to only 6% and 4% in multiple sclerosis and movement disorders, respectively |
| 11 | PR | There were significant differences between the four neurological conditions regarding whether the neurologist perceived follow-ups by telephone as satisfactory. Telephone consultations were viewed as better suited for epilepsy care than multiple sclerosis (P = 0.033) or movement disorders (P < 0.001). |
| 11 | PR | Headache was viewed as better suited for telephone consultations than multiple sclerosis (P = 0.017) and movement disorders (P = 0.002). |
| 11 | PR | However, no differences were reported between epilepsy and headache. Follow-up teleconsultations for multiple sclerosis were reported as better suited than movement disorders (P < 0.002). |
| 11 | PR | Neurologists within epilepsy care were more comfortable in making changes in medication dosage by telephone than neurologists responsible for movement disorder patients |
| 11 | PR | Reduced standard of care during the initial phase of the pandemic was reported by fewer neurologists within epilepsy or headache care than in multiple sclerosis (P < 0.001) or movement disorders (P < 0.001). |
| 11 | PR | There was no significant difference between the reported standard of epilepsy and headache care. |
| 11 | PR | The proportion of participants who reported that telephone consultations did not work satisfactory for the patients, and the corresponding proportion that reported an overall reduced standard of care during the pandemic were, respectively, 9% and 30% in epilepsy, 3% and 42% in headache, 24% and 71% in multiple sclerosis, and 57% and 74% in movement disorders. |
| 12 | PR | We specifically asked about first visits: of the respondents, 42.4% performed first visits also with telemedicine, while a significant percentage (38%) preferred to postpone them to see the patients onsite after the end of the lockdown period. |
| 12 | PR | Care provided during the telemedicine visit was appropriate and enough in the majority of patients. |
| 12 | PR | Half of the epileptologists reported that they rarely had to reschedule the patient for an onsite follow-up. |
| 12 | PR | The most frequent reason to schedule another (onsite) visit was the need for blood work, (electroencephalogram (EEG), or neuroimaging to decide about diagnosis or treatment.) |
| 12 | PR | The most frequent reason to schedule another (onsite) visit was the need for blood work, electroencephalogram (EEG), or neuroimaging to decide about diagnosis or treatment. |
| 12 | PR | The next most frequently reported reason for rescheduling (21%) was the need to perform a neurological examination onsite |
| 12 | PR | Most neurologists (90%) had to provide over the phone information regarding results of EEG or neuroimaging tests, and most believed that the patients understood it correctly. |
| 12 | PR | Most neurologists (90%) had to provide over the phone information regarding results of EEG or neuroimaging tests, (and most believed that the patients understood it correctly). |
| 12 | PR | We also assessed if other types of sensitive information were provided to the patients. Of all respondents, 56.1% provided information about pregnancy risks over the phone in women of childbearing potential, while 43.9% felt that type of information should be given during an onsite visit. |
| 12 | PR | Regarding sudden expected unrelated death in epilepsy (SUDEP), the great majority (84.4%) reported that they preferred that discussion to be held with the patient and family during a normal visit. |
| 12 | PR | Almost 60% of the respondents had an epilepsy surgery program in their hospital. Only 27.4% of them had to explain the results of the surgical evaluation over the phone. Of those, only 62.5% felt the patient had understood the information correctly. |
| 12 | PR | A majority of the responders (83.3%) reported not having administered any questionnaires (quality of life, adverse effects, depression, etc.) over the phone or by videoconference. |
| 12 | PR | Almost all the participants had the electronic medical record used by their hospitals available while they contacted their patients; however, 30.3% reported not having access to the common electronic medical record (the one containing all medical information of one patient, including data from different medical centers). Almost 20% could not make electronic preprescriptions fromtheir computers at the time they attended the patients. |
| 12 | PR | When asked about patients' impressions, 78.8% of respondents stated that some patients would like to have this type of telephone/videoconference follow-up in the future. |
| 12 | PR | When asked about patients' impressions, ….Only 10% felt that very few patients would be interested in this system. |
| 13 | PR | 44.1% of the experts were able to carry out neurological examinations, although the majority were not able to evaluate the sensitivity and visual test. |
| 13 | PR | For assessing MS disability by telemedicine, only 38.7% advise and continue using the conventional Expanded Disability Status Scale (EDSS) via video call (Tele-EDSS). |
| 13 | PR | On the other hand, 48.6% of experts are currently using Patient Determined Disease Steps (PDDS) to replace EDSS in remote examination. |
| 13 | PR | Only 9.9% are using Symbol Digit ModalitiesTest (SDMT) as a cognitive test that can be delivered remotely. |
| 13 | PR | Regarding relapse, 85.6% of the experts believe they are able to identify a relapse via telemedicine, and 52.3% advised to treat directly with oral corticoids without the necessity of a face-toface appointment. |
| 14 | PR | Participants were asked to compare telemedicine to in-person visits among multiple facets of the patient evaluation (i.e., history taking, examination, imaging review, diagnosis). Analysis of the mean Likert scale responses demonstrated an overall leftward skew (worse or slightly worse) of provider opinion toward telemedicine evaluation |
| 14 | PR | Most respondents felt that telemedicine was at least equivalent to in-person visits for communicative tasks, such as taking a patient history 63.4% |
| 14 | PR | (Most respondents felt that telemedicine was at least equivalent to in-person visits for communicative tasks such as ) reviewing and explaining imaging (72.3%), |
| 14 | PR | "Most respondents felt that telemedicine was at least equivalent to in-person visits for communicative tasks such as " formulating and communicating a treatment plan (67.7%). |
| 14 | PR | Respondents had significantly less confidence in the physical exam portions of the evaluation |
| 14 | PR | (Respondents had significantly less confidence) telemedicine was worse or much worse in assessing neurologic deficits (94.6%), provocative testing (89.5%) and myelopathy (89.0%) |
| 14 | PR | In almost all analyses, region was not associated with differences in participant confidence in telemedicine |
| 14 | PR | participant age was not found to be associated with confidence in the telemedicine evaluation |
| 14 | PR | However, multivariate adjustment demonstrated that participants > 55 years old were more likely to believe telemedicine was equivalent or better to in-person visits for the assessment of spinal deformity |
| 14 | PR | Compared with telephone (audio only), the use of videoconferencing technology was associated with increased confidence in the ability of telemedicine to formulate and communicate a treatment plan when compared to in-person visits |
| 14 | PR | Providers who had performed > 50 telemedicine visits demonstrated increased confidence in the ability of telemedicine to formulate and communicate a treatment plan and make an accurate diagnosis |
| 14 | PR | On multivariate analysis, respondents experienced with telemedicine were more likely to believe telemedicine was equivalent or better than in-person visits in the ability to make an accurate diagnosis |
| 14 | PR | those with neurosurgery training described increased confidence in telemedicine for imaging review and explanation |
| 14 | PR | Our anchor question for provider trust in telemedicine revealed that most respondents (74.9%) believed that the initial visit could be performed through telemedicine. |
| 14 | PR | An affirmative response to the anchor question was associated with slightly increased confidence in telemedicine in almost all facets of the patient evaluation, with the exception of the assessment of myelopathy and imaging review. (However, none of these relationships were sustained in the multivariate analyses.) |
| 15 | PR | While 62% of providers agreed that a telehealth visit was more convenient for them than an inperson visit, |
| 15 | PR | only 42% agreed that they preferred telehealth to in-person visits. |
| 15 | PR | Sixty-two percent of providers agreed that they were able to adequately address patient’s needs using telehealth |
| 15 | PR | but 52% of providers disagreed that they were able to do the relevant neurological examination virtually |
| 15 | PR | Eighty-three percent of providers believed that telehealth was a safe and effective method of seeing patients. |
| 15 | PR | Eighty-two percent of providers felt their communication with their patients was adequate when using telehealth |
| 15 | PR | 85% of providers agreed that they spent enough time with their patient |
| 15 | PR | Eighty-two percent of providers stated that they would like to do more telehealth visits in the future, |
| 15 | PR | with 85% responding that they wished to incorporate telehealth in their future practice. |
| 15 | PR | When asked what type of visit telehealth was most suited for, 62% of providers chose follow-up visits, 18% chose first visits, 10% chose presurgery visits, and 10% chose postsurgery visits. |
| 15 | PT | Follow-up visits were reported to be 38% of patient telehealth visits, with the next most common visit being first visits (26%). |
| 15 | PT | Ninety-two percent of patients agreed that they were satisfied with their telehealth visit |
| 15 | PT | Sixtynine percent of patients agreed that they were able to join the telehealth video call easily. |
| 15 | PT | Ninety percent of patients agreed that their provider spent enough time with them during the telehealth visit, |
| 15 | PT | 91% of patients agreed that their provider satisfactorily addressed their clinical needs |
| 15 | PT | Sixty-four percent of patients agreed that they were able to adequately show their clinical signs to their provider, |
| 15 | PT | while 72% agreed that their provider was able to sufficiently explain radiological findings to them over telehealth. |
| 15 | PT | Eighty-eight percent of patients agreed that their telehealth visit was more convenient for them than an in-person visit. |
| 15 | PT | Only 46% of patients agreed that they preferred telehealth visits to in-person visits, |
| 15 | PT | with another 40% stating they were neutral on the matter (telehealth visits to in-person visits). |
| 15 | PT | Similarly, only 36% of patients stated they would like their future visits to be telehealth visits, |
| 15 | PT | with 48% patients stating they felt neutrally about this statement (would like their future visits to be telehealth visits). |
| 16 | PR | More than 90% of participants reported using an EMR. |
| 16 | PR | 73.9% of users plan to continue video visits in their practice after public health emergency, whereas 17.6% are unsure. |
| 16 | PR | During the COVID-19 pandemic, 32.2% of survey participants (30.7% US and 38.9% non-US, P = 0.43) performed remote testing interpretation. |
| 16 | PR | One participant noted that in-person testing was not being performed at their institution during the pandemic, which limited the opportunity. |
| 16 | PR | Ophthalmic imaging and visual fields remained the main types of tests being interpreted without seeing the patient. |
| 16 | PR | During COVID-19, 15.3% (14.4% US and 19.4% non-US) of participants used online second opinions and 18.7% (18.6% US and 19.4% non-US) used e-consults. |
| 16 | PR | Conditions for which >50% of participants indicated video visits are helpful were migraine with aura (65.0%). |
| 16 | PR | Conditions for which >50% of participants indicated video visits are helpful were …. pituitary tumor with prior visual fields... |
| 16 | PR | Conditions for which >50% of participants indicated video visits are helpful were …. MRI results (62.1%). |
| 16 | PR | Conditions for which >50% of participants indicated that video visits are not helpful were nonarteritic anterior ischemic optic neuropathy (63.4%), possible arteritic ischemic optic neuropathy (60.5%), and optic atrophy (70.8%). |
| 17 | PT | A delay in performance of epilepsy-related tests occurred in 37 patients (14.5%). Routine EEG was the test most often delayed (n = 11; 29.7%), followed by MRI (n = 9; 24.3%) and video-EEG monitoring (n = 8; 21.6%). |
| 17 | PT | Seven patients (2.7%) reported difficulties obtaining their medication supply in the pharmacy, six (85.7%) because their prescription had expired and one (14.3%) because the medication was not available. |
| 17 | PT | Confinement-related anxiety and depression were reported by 68 (26.7%) and 22 (8.6%) patients, respectively, and 31 patients (12.2%) reported having both anxiety and depression symptoms since the start of confinement. Regarding sleep disturbance, 72 patients (28.2%) reported insomnia and 22 patients (8.6%) reported that their usual sleep schedules had changed. Eighteen patients (7.1%) stated that they were sleeping for a longer period per day than usual due to the confinement measures |
| 17 | PT | Seventy-three patients (28.6%) stated that the usual family income had decreased because of the confinement-related restrictions |
| 17 | PT | 90 patients (35.3%) answered that their worst fear was contracting COVID-19 infection. |
| 17 | PT | Several factors were associated with increased seizure frequency during the confinement period.Probability of increased seizure frequency depending on the cumulative association of 5 risk factors (tumor-related etiology, drug-resistant epilepsy, fear for epilepsy, insomnia, and reduction of economic income). |
| 17 | PT | When asked about their perception of the usefulness of telephone visits during confinement, 124 patients (48.6%) stated they were very satisfied, and 90 (35.3%) were quite satisfied |
| 17 | PT | Seven patients (2.7%) reported that the visits were unsatisfactory, and some refused to answer (n = 34; 13.3%). None of the clinical factors were predictive of a better opinion of telemedicine in this setting. |
| 17 | PT | For future follow-ups, 97 (38%) patients considered it useful to conduct telephone visits, |
| 17 | PT | For future follow-ups, while 93 (36.5%) preferred face-to-face visits. |
| 17 | PT | For future follow-ups, Fifty-five patients (21.6%) said they did not prefer one of these options over the other. |
| 17 | PT | Interestingly, five patients (2%) spontaneously mentioned that they would like to try a video call option. |
| 17 | PT | In the analysis of clinical factors potentially predictive of a better perception of telemedicine in the future, patients with a greater fear for COVID-19 felt positive about conducting telephone visits |
| 17 | PT | A non-significantly higher percentage of patients with drug-resistant epilepsy preferred face-to-face visits. |
| 18 | PT | Patients in the 70–79 age bracket had the largest percentage of telemedicine appointments (36.5%), followed by patients in the 60–69 age bracket (27.2%). |
| 18 | PT | Age distribution did not differ between inclinic and telemedicine visits; hence, age did not play a role in whether patients established a telemedicine visit (p = 0.17). |
| 18 | PT | Men were more likely than women to transition to a telemedicine visit (p = 0.02) |
| 18 | PT | The majority of patients evaluated by telemedicine were Caucasian (72.9%), followed by Other (12.5%), Black (11.7%), and Asian (2.92%). |
| 18 | PT | The percentage of telemedicine compared to in-person visits prepandemic increased for Caucasians by 4%, while the percentage decreased by 3% for Blacks. |
| 18 | PT | On average, patients who participated in telemedicine were located farther from the clinic than the population of patients evaluated in person over the last year. |
| 18 | PT | Only age revealed a statistically significant shift towards telephone visits compared to other factors, such as race, distance, and diagnosis; older patients preferred telephone visits compared with audio-video visits |
| 18 | PT | This suggests that patients with DBS were more likely to convert their visit type to telemedicine when asked by schedulers compared to patients without DBS. |
| 18 | PT | We further analyzed the distribution of diagnoses conducted among the audiovisual vs. telephone visits, we did not identify statistically significant differences. |
| 18 | PT | Press Ganey results were obtained for all providers (n = 19). The average Press Ganey score prepandemic (September 1, 2019 to March 16, 2020) was 89.6% (n = 974) vs. 92.9% (n = 113) during the COVID-19 pandemic, demonstrating that patient satisfaction during telemedicine encounters was not compromised during the pandemic. |
| 18 | PR | In the two surveys, approximately 83–89% of clinicians were either satisfied or very satisfied with telemedicine visits. |
| 19 | PT | The reasons for connection failure were the lack of PC, tablet, or phone with Internet connection in 8 cases (23.5%) |
| 19 | PT | The reasons for connection failure were ...the difficulty in establishing a connection in 26 cases (76.4%). |
| 19 | PT | No significant differences were observed among the two groups (success vs failed video vist) concerning age, gender, education, and Mini Mental State Examination (MMSE) from previous in person visit |
| 19 | PT | Televisits performed in the presence of subjects of younger generation had a successful rate higher than the group without younger generation caregiver |

Study # references:

1. Courtney E, Blackburn D, Reuber M. Neurologists’ perceptions of utilising tele-neurology to practice remotely during the COVID-19 pandemic. Patient Educ Couns. 2021;104(3):452-9.
2. McKenna M C, Al-Hinai M, Bradley D, Doran E, Hunt I, Hutchinson S, et al. Patients’ experiences of remote neurology consultations during the COVID-19 pandemic. Eur Neurol. 2020;83(6):622-5.
3. Casares M, Wombles C, Skinner HJ, Westerveld M, Gireesh ED. Telehealth perceptions in patients with epilepsy and providers during the COVID-19 pandemic. Epileps Behav. 2020. doi:10.1016/j.yebeh.2020.107394.
4. von Wrede R, Moskau-Hartmann S, Baumgartner T, Helmstaedter, C, Surges, R. Counseling of people with epilepsy via telemedicine: experiences at a German tertiary epilepsy center during the COVID-19 pandemic. Epilepsy Behav. 2020. doi:10.1016/j.yebeh.2020.107298.
5. Willems LM, Balcik Y, Noda AH, Siebenbrodt K, Leimeister S, McCoy J, et al. SARS-CoV-2-related rapid reorganization of an epilepsy outpatient clinic from personal appointments to telemedicine services: A German single-center experience. Epilepsy Behav. 2020. doi:10.1016/j.yebeh.2020.107483.
6. Smith M, Nakamoto M, Crocker J, Morden FT, Liu K, Ma E, et al. Early impact of the COVID‐19 pandemic on outpatient migraine care in Hawaii: Results of a quality improvement survey. Headache. 2021;61(1):149-56.
7. Saliba-Gustafsson EA, Miller-Kuhlmann R, Kling SMR, Garvert, DW, Brown-Johnson CG., Lestoquoy, AS, et al. Rapid implementation of video visits in neurology during COVID-19: mixed methods evaluation. JMIR. 2020. doi:10.2196/24328.
8. Harper K, Roof M, Wadhawan N, Terala A, Turchan M, Bagnato F, et al. Vanderbilt University Medical Center Ambulatory Teleneurology COVID-19 Experience. Telemed E Health. 2021;27(6):701-5.
9. Ryu WHA, Kerolus MG, Traynelis VC. Clinicians’ user experience of telemedicine in neurosurgery during COVID-19. World Neurosurg. 2021. doi:10.1016/j.wneu.2020.10.101.
10. Kristoffersen ES, Sandset EC, Winsvold BS, Faiz KW, Storstein AM. Experiences of telemedicine in neurological out‐patient clinics during the COVID‐19 pandemic. Ann Clin Transl Neurol. 2021;8(2):440-7.
11. Conde-Blanco E, Centeno M, Tio E, Muriana D, García-Peñas JJ, Serrano P, et al. Emergency implementation of telemedicine for epilepsy in Spain: results of a survey during SARS-CoV-2 pandemic. Epilepsy Behav. 2020. doi:10.1016/j.yebeh.2020.107211.
12. Alonso R, Carvajal R, Boaventura M, Galleguillos L. Experience of South American MS and/or NMOSD experts in practice during the COVID-19 pandemic: focus on telemedicine. Mult Scler Relat Disord. 2021. doi:10.1016/j.msard.2020.102702.
13. Lovecchio F, Riew G J, Samartzis D, Louie PK, Germscheid N, An HS, et al. Provider confidence in the telemedicine spine evaluation: results from a global study. Eur Spine J. 2021;30:2109-23.
14. Mohanty A, Srinivasan VM, Burkhardt JK, Johnson J, Patel AJ, Sheth SA, et al. Ambulatory neurosurgery in the COVID-19 era: patient and provider satisfaction with telemedicine. Neurosurg Focus. 2020. doi:10.3171/2020.9.FOCUS20596.
15. Fonseca E, Quintana M, Lallana S, Restrepo, JL, Abraira L, Santamarina E, et al. Epilepsy in time of COVID‐19: a survey‐based study. Acta Neurol Scand. 2020;142(6):545-54.
16. Esper C D, Scorr L, Papazian S, Bartholomew D, Esper GJ, Factor SA. Telemedicine in an academic movement disorders center during COVID-19. J Mov Disord. 2021. doi:10.14802/jmd.20099.
17. Arighi A, Fumagalli GG, Carandini T, Pietroboni AM, De Riz MA, Galimberti D, et al. Facing the digital divide into a dementia clinic during COVID-19 pandemic: caregiver age matters. Neurol Sci. 2021;42(4):1247-51
18. Chesnel C, Hentzen C, Le Breton F, Turmel N, Tan E, Haddad R, Amarenco G. Efficiency and satisfaction with telephone consultation of follow‐up patients in neuro‐urology: Experience of the COVID‐19 pandemic. Neurourol Urodyn. 2021;40(3):929-37.
19. Kummer BR, Sweetnam C, Vickrey BG, Naasan G, Harvey D, Gallagher K, et al. Teleneurology Expansion in Response to the COVID-19 Outbreak at a Tertiary Health System in New York City. Neurol Clin Pract. 2021. doi:10.1212/CPJ.0000000000001057
